# Shiny-Calorie: a context-aware application for indirect calorimetry data analysis and visualization using R

**DOI:** 10.1093/bioadv/vbaf270

**Published:** 2025-10-29

**Authors:** Stephan Grein, Tabea Elschner, Ronja Kardinal, Johanna Bruder, Akim Strohmeyer, Karthikeyan Gunasekaran, Jennifer Witt, Hildigunnur Hermannsdóttir, Janina Behrens, Mueez U-Din, Jiangyan Yu, Gerhard Heldmaier, Renate Schreiber, Jan Rozman, Markus Heine, Ludger Scheja, Anna Worthmann, Joerg Heeren, Dagmar Wachten, Kerstin Wilhelm-Jüngling, Alexander Pfeifer, Jan Hasenauer, Martin Klingenspor

**Affiliations:** Life and Medical Sciences (LIMES) Institute and Bonn Center for Mathematical Life Sciences, University of Bonn, Bonn 53113, Germany; Institute for Cardiovascular Sciences University Hospital, University of Bonn, Bonn 53113, Germany; Institute for Neurovascular Cell Biology University Hospital, University of Bonn, Bonn 53113, Germany; Institute of Innate Immunity, Medical Faculty, University of Bonn, Bonn 53113, Germany; EKFZ for Nutritional Medicine, Technical University of Munich, Freising-Weihenstephan 85354, Germany; EKFZ for Nutritional Medicine, Technical University of Munich, Freising-Weihenstephan 85354, Germany; Department of Biochemistry and Molecular Cell Biology, University Medical Center Hamburg-Eppendorf, Hamburg 20246, Germany; Department of Biochemistry and Molecular Cell Biology, University Medical Center Hamburg-Eppendorf, Hamburg 20246, Germany; Molecular Nutritional Medicine, TUM School of Life Sciences, Technical University of Munich, Munich, Freising 85354, Germany; Department of Biochemistry and Molecular Cell Biology, University Medical Center Hamburg-Eppendorf, Hamburg 20246, Germany; Turku PET Centre, University of Turku and Turku University Hospital, Turku, Turku 20520, Finland; Institute of Clinical Genetics and Genomic Medicine, University Hospital Würzburg, Würzburg 97070, Germany; Animal Physiology, Department of Biology, Marburg University, Marburg, Marburg 35037, Germany; Institute of Molecular Biosciences, University of Graz, Graz 8010, Austria; Luxembourg Centre for Systems Biomedicine, University of Luxembourg, Esch-Belval 4001, Luxembourg; Department of Biochemistry and Molecular Cell Biology, University Medical Center Hamburg-Eppendorf, Hamburg 20246, Germany; Department of Biochemistry and Molecular Cell Biology, University Medical Center Hamburg-Eppendorf, Hamburg 20246, Germany; Department of Biochemistry and Molecular Cell Biology, University Medical Center Hamburg-Eppendorf, Hamburg 20246, Germany; Department of Biochemistry and Molecular Cell Biology, University Medical Center Hamburg-Eppendorf, Hamburg 20246, Germany; Institute of Innate Immunity, Medical Faculty, University of Bonn, Bonn 53113, Germany; Institute for Cardiovascular Sciences University Hospital, University of Bonn, Bonn 53113, Germany; Institute for Neurovascular Cell Biology University Hospital, University of Bonn, Bonn 53113, Germany; Institute of Pharmacology and Toxicology, University Hospital, University of Bonn, Bonn 53115, Germany; Life and Medical Sciences (LIMES) Institute and Bonn Center for Mathematical Life Sciences, University of Bonn, Bonn 53113, Germany; EKFZ for Nutritional Medicine, Technical University of Munich, Freising-Weihenstephan 85354, Germany; Molecular Nutritional Medicine, TUM School of Life Sciences, Technical University of Munich, Munich, Freising 85354, Germany; ZIEL – Institute for Food & Health, Technical University of Munich, Freising 85354, Germany

## Abstract

**Motivation:**

Indirect calorimetry is the standard method for metabolic phenotyping of animal models in pre-clinical research, supported by mature experimental protocols and widely used commercial platforms. However, a flexible, extensible, and user-friendly software suite that enables standardized integration of data and metadata from diverse metabolic phenotyping platforms—followed by unified statistical analysis and visualization—remains absent.

**Results:**

We present Shiny-Calorie, an open-source interactive application for transparent data and metadata integration, comprehensive statistical data analysis, and visualization of indirect calorimetry datasets. Shiny-Calorie supports the majority of standard data formats across commercial metabolic phenotyping platforms, such as TSE and Sable Systems, COSMED platform and CLAMS/Columbus instruments, and provides export functionality of processed data into standardized formats. Built using GNU R with a reactive interface, Shiny-Calorie enables intuitive exploration of complex, multi-modal longitudinal datasets comprising categorical, continuous, ordinal, and count variables. The platform incorporates state-of-the-art statistical methods for robust hypothesis testing, thereby facilitating biologically meaningful interpretation of energy metabolism phenotypes, including resting metabolic rate and energy expenditure. Together, these features, streamline routine analysis workflows and enhances reproducibility and transparency in metabolic phenotyping studies.

**Availability and implementation:**

Shiny-Calorie is freely available at https://shiny.iaas.uni-bonn.de/Shiny-Calorie/. User documentation and source code are available at https://github.com/ICB-DCM/Shiny-Calorie. A docker image is available from https://hub.docker.com/r/stephanmg/Shiny-Calorie. Instructional screen recordings are available on https://www.youtube.com/@shiny-calorie.

## 1 Introduction

Indirect calorimetry (IC) is an indispensable tool for metabolic phenotyping (Even and Nadkarni 2012), and routinely used in pre-clinical and clinical research particularly in the domains of adipose tissue (Din *et al.* 2006, Fischer *et al.* 2019, Dieckmann *et al.* 2022) and nutritional medicine (Klingenspor *et al.* 2020). IC is used to quantify both variations and mean differences in energy expenditure by tracking the respiratory gases—oxygen and carbon dioxide. These measurements enable the analysis of correlations and effect sizes related to genotype or diet stratifications, typically corrected for variations in body composition. IC has been utilized to identify and characterize metabolic phenotypes for decades (Ravussin *et al.* 1986, Weinsier 2002, Bjursell *et al.* 2008). It also allows for the calculation of resting metabolic rates, which are especially relevant in the context of phenotyping in obesity research (Wang *et al.* 2021, Dieckmann *et al.* 2022).

Several commercial and academic software packages exist for the processing and visualization of IC data, such as the proprietary analysis tools provided by TSE and Sable Systems, as well as standalone packages tailored to specific institutional workflows, e.g. CalR (Mina *et al.* 2018), respR (Harianto *et al.* 2019), and CaloPy (Loipfinger *et al.* 2025). Yet, these existing solutions typically focus on individual experiments, and poorly support cross-study analyses, and offer limited extensibility or reproducibility. In most cases, metadata must be manually curated, and labels are not harmonized across datasets, complicating efforts to conduct joint analyses across cohorts or institutions. For this reason, many users of IC have in the past written there own R and Python scripts, lacking user-friendly interfaces and support for harmonized metadata integration.

**Figure 1.**
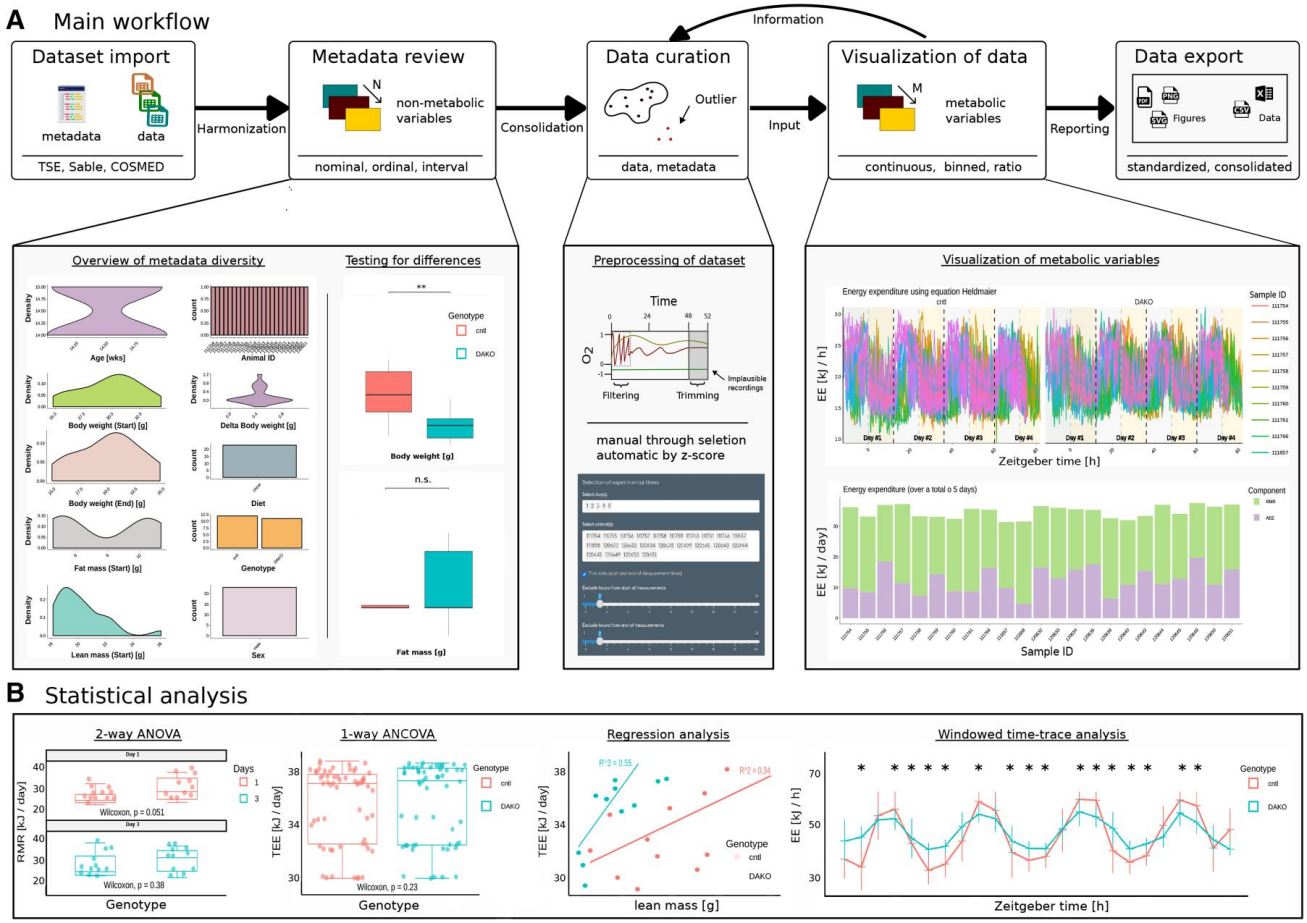
Illustration of Shiny-Calorie workflow and features. (A) Dataset import, metadata review, data curation, visualization of data and data export. Exemplary visualizations for different datasets are shown, i.e. bar, density, box, silhouette, and time-series plots. (B) Overview of statistical analysis methods and corresponding visualization, including statistical analysis with ANOVA and ANCOVA, regression analysis, and time-series analysis.

Here, we present the Shiny-Calorie application, allowing seamless integration of data and harmonization of metadata, enabling context-aware data visualization and analysis, using a streamlined user-interface, addressing the aforementioned limitations. The application supports many common file formats (cf. Table 3, available as supplementary data at *Bioinformatics Advances* online, noteworth to mention concurrent support of CalR and CaloPy files) and was developed in collaboration with experienced IC researchers toward their requirements for data analysis and visualization requirements. Quantification of energy expenditure (EE), resting metabolic rate (RMR), and activity-dependent energy expenditure (AEE) and linking of results with metadata, i.e. genotype, food and water intake, physical activity, etc. (Fig. 1; Figs S1–S3, available as supplementary data at *Bioinformatics Advances* online) is available. Implemented in the GNU R ecosystem, Shiny-Calorie provides access to state-of-the-art visualization and statistical analysis tools. Publication-ready high-quality figures and standardized data export of computed and consolidated datasets are provided. Shiny-Calorie is available as (i) a platform-independent web application running in common web browsers (applicable when users lack administrator privileges or do not want to install software), (ii) a standalone Desktop application (applicable when connectivity is limited), and (iii) a Docker container (promoting a quick adoption by users and instant transferability of workflows between workstations).

## 2 Feature overview

Shiny-Calorie offers a comprehensive set of features for the integration, processing, analysis, and visualization of IC data. The following sections provide a detailed description of the software’s key capabilities, organized according to the typical user workflow. Advanced features and extended options are illustrated in the Supporting Information (SI) and demonstrated through representative use-cases.

### 2.1 Dataset import

Shiny-Calorie enables the import of datasets from multiple metabolic phenotyping platforms, including TSE Systems (LabMaster, PhenoMaster), PhenoSys (Calobox), Sable Systems, COSMED platforms (Generic and QNRG), and Columbus instruments CLAMS Oxymax. Additionally read-in of data export from CalR (Mina *et al.* 2018) and CaloPy (Loipfinger *et al.* 2025) are supported. Supported file formats and versions are listed in Table 1, available as supplementary data at *Bioinformatics Advances* online. At a minimum, imported datasets must contain an Animal ID column and metabolic variables such as $O_{2}$ and $CO_{2}$, typically recorded at regular time intervals. Shiny-Calorie accepts raw exports directly from metabolic phenotyping platforms and can ingest data files containing metadata either in headers or in structured external files. Moreover, Shiny-Calorie also supports ontology-based hierarchical metadata via a standardized Metadata sheet (Seep *et al.* 2024) for enhanced data annotation and interoperability.

### 2.2 Metadata review

Metadata in Shiny-Calorie can be supplied in two ways: either manually, through user input during the data review process, by uploading a standardized Metadata sheet or through an auxiliary Shiny app assisting with metadata collection. The Metadata sheet enables structured annotation of datasets with experimental conditions, treatments, photoperiod, and other contextual variables. Even if metadata are imported, the user can manually change the metadata, e.g. to correct errors or to fill gaps. Once metadata is provided—either via manual input or upload—Shiny-Calorie performs automatic harmonization of metadata labels across datasets. This includes consolidating variable names, resolving inconsistencies, and encoding the metadata into a standardized internal format to support joint analysis across multi-cohort studies. Harmonization reduces the risk of errors from inconsistent labeling and facilitates reproducible downstream analysis.

### 2.3 Data curation

Shiny-Calorie provides robust tools for the cleaning and curation of IC datasets to ensure data quality and consistency prior to downstream analysis. To reach data integrity, users may enable a set of pre-defined consistency checks via checkboxes during the metadata review step. Identification of negative values in respiratory gas exchange measurements, filtering of high-frequency data points, enforcement of complete days, and validation of temperature records are possible.

After data upload, the consistency checks are automatically applied to detect these common anomalies in the raw measurements. Users are notified of issues and can decide on appropriate corrective actions. Outliers can be interactively removed using a graphical rectangular/lasso selection, or automatically through an adjustable z-score thresholding.

Individual subjects and days can be excluded from the dataset based on metadata attributes or statistical outlier detection. Additionally, grouping and filtering based on any categorical metadata variable (e.g. genotype, treatment, diet) are supported to facilitate stratified and comparative analyses.

To eliminate artifacts introduced during the initial habituation phase or final handling of subjects, users can trim time segments from the beginning and end of experiments. Reliable measurement periods, such as intermediate full days, can be selected using drop-down menus. Selection can be performed either based on zeitgeber time or conventional calendar dates. Photoperiod information is inferred either from the Metadata sheet or specified manually.

### 2.4 Data processing and visualization

Shiny-Calorie reconstructs energy expenditure (EE) from raw IC data using standard equations, including the Heldmaier equation (Heldmaier 1975), $EE\;[\text{kJ}/h]=(4.44*1.43*\mathrm{RER})*\dot{V}O_{2}\;[\text{ml}/h]*\frac{3.6}{1000}$ where $\mathrm{RER}=\frac{\dot{V}CO_{2}\;[\text{ml}/h]}{\dot{V}O_{2}\;[\text{ml}/h]}$. Alternative equations are supported (cf. Table 2, available as supplementary data at *Bioinformatics Advances* online). Derivation of secondary quantities such as RMR and AEE, using methods based on the variability of $O_{2}$/$CO_{2}$ signals are available. When physical activity data is available, Shiny-Calorie generates locomotion density maps and behavioral summaries for experimental validation.

Derived metrics are calculated to assess substrate utilization (e.g. fat versus carbohydrate oxidation). Visualizations of raw and reference $O_{2}$/$CO_{2}$ signals assist in evaluating data quality and validating experimental conditions. Shiny-Calorie supports the plotting of all available variables, such as food/water intake, locomotor activity and photoperiods. Windowed time-trace analyses allow for exploration of temporal dynamics (e.g. hourly RMR/EE profiles), enabling detection of time-specific differences between groups.

### 2.5 Statistical analysis

Shiny-Calorie integrates standard statistical methods for hypothesis testing. Available models include multi-way ANOVA, ANCOVA, and generalized linear models, with support for variable number of comparison groups. Covariate correction (e.g. for body weight) is available. Diagnostic tools and assumption tests (e.g. Shapiro–Wilk, Levene’s test) are provided to verify prerequisites for parametric testing. Post-hoc comparisons (with correction for multiple testing) and statistical summaries are generated, and test assumption results are summarized in a binary format (check marks), facilitating interpretation for users without statistical expertise. Statistical significance is reported using conventional asterisk notation.

### 2.6 Data export

All processed and derived data can be exported in multiple formats. Visualizations are available in vector or bitmap formats and as interactive plots (HTML). Data tables can be exported as CSV files, and consolidated multi-cohort datasets in Excel or CalR-compatible formats. Exported data can be used in downstream analysis and integration into (offline) computational workflows, facilitate reproducibility, and allow cross-study comparisons, available via the Export section.

## 3 Dissemination, training, and use-cases

To support dissemination, training, and user adoption, Shiny-Calorie is accompanied by a comprehensive suite of instructional resources and real-world examples.

**Documentation:** Extensive documentation of Shiny-Calorie’s functionalities, including API references and deployment instructions, is available on the project’s documentation page: https://ICB-DCM.github.io/Shiny-Calorie.

**Use-cases:** Representative use-cases are provided in the Supporting Information (SI) to demonstrate Shiny-Calorie’s capabilities in real-world scenarios, including metadata preparation, statistical analysis and visualization, and wavelet analysis. Example datasets are bundled with the application and directly loadable.

**Video Materials:** Instructional screen recordings are available on YouTube: http://youtube.com/@Shiny-Calorie. The application includes an interactive guide (accessible via the Guide button) and context-specific help sections (accessible via the Help button). These resources are designed to facilitate onboarding and self-paced training.

**Training-Oriented Design:** Analysis workflows were developed in close collaboration with experimental partners to optimize usability for routine laboratory applications. Design decisions prioritize transparency, reproducibility, and ease of use, ensuring that even non-specialist users can efficiently perform and interpret metabolic analyses.

## 4 Implementation and availability

Shiny-Calorie is implemented as a web-based application using the Shiny framework within the GNU R ecosystem under the BSD-3-Clause license. Shiny-Calorie is available as:

- **Web Application:** Hosted on institutional infrastructure and accessible via web browsers (no installation required) (https://shiny.iaas.uni-bonn.de/Shiny-Calorie).
- **Docker Image:** Available as an OCI-compliant container for rapid deployment and reproducible analysis workflows (https://hub.docker.com/r/stephanmg/Shiny-Calorie).
- **Standalone Desktop Installer:** Provided via Github for offline use on local computers (https://github.com/ICB-DCM/Shiny-Calorie-Wrapper).

The source code and API reference are available on Github (https://github.com/ICB-DCM/Shiny-Calorie).

## 5 Discussion

Shiny-Calorie addresses a key bottleneck in the field of metabolic phenotyping by enabling harmonized, reproducible, and interactive analysis of IC datasets across multiple platforms and experimental contexts. Its modular and extensible architecture supports both single-study and multi-cohort analyses, enhancing the utility of IC data in preclinical and translational research.

By automating data harmonization and integrating robust statistical and visualization methods into a single platform, Shiny-Calorie substantially reduces the overhead of metadata curation and manual processing. It supports stratified analysis of metabolic parameters (e.g. EE, RMR, AEE) with or without physical activity data, making it adaptable to a wide range of experimental designs and instrumentation configurations.

Shiny-Calorie’s design reflects the practical needs of both computational analysts and wet-lab researchers. Through an intuitive graphical interface and guided workflows, it empowers users with varying levels of technical expertise to perform high-quality metabolic data analyses. The platform’s open-source nature and multi-platform availability foster transparency, reproducibility, and collaborative development.

In summary, Shiny-Calorie contributes as a broadly applicable and user-centered solution to the metabolic research community, supporting standardized, scalable, and insightful interrogation of energy metabolism in diverse biological systems.

## Supplementary Material

vbaf270_Supplementary_Data

## Data Availability

No new data were generated. Example data sets for downloadable through the application and deposited in the corresponding Github repository.
