# Supplementary material for: Shiny-Calorie: a context-aware application for indirect calorimetry data analysis and visualization using R
Source: Bioinform Adv. 2025 Oct 29;6(1):vbaf270. doi: 10.1093/bioadv/vbaf270 (PMC12867577; doi:10.1093/bioadv/vbaf270)
Supplement: vbaf270_Supplementary_Data [file vbaf270_supplementary_data.zip › 15-Jan-2026_092528_Calo_Shiny-Supplemental.pdf]

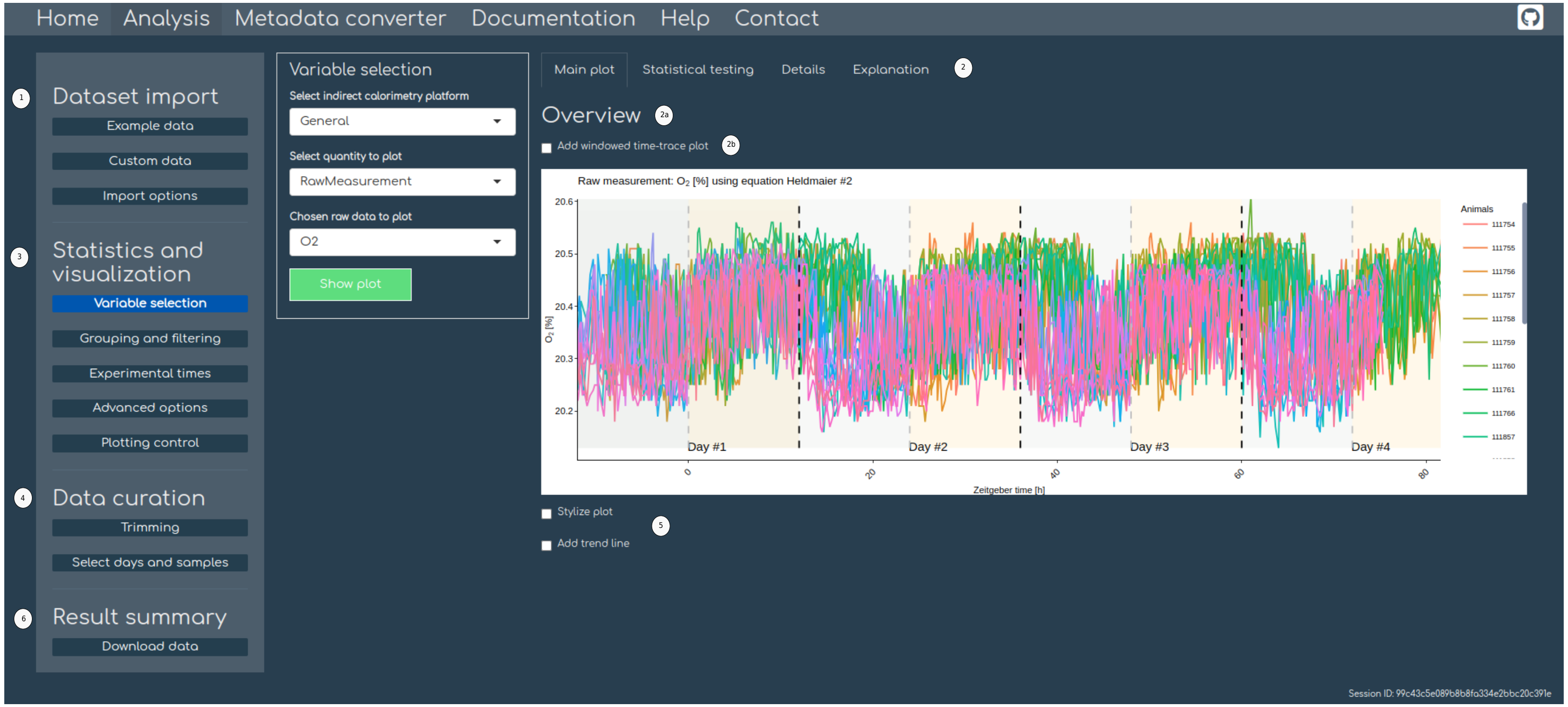

Supplementary Figure S1: **Overview of Shiny-Calorie's user interface.** **1** Dataset import: The section allows to provide own datasets and metadata, or use one of the provided built-in data sets. A user guide is available from the help content in the navigation bar. Under the subsection `Example data` one finds a Ciceron guide which will walk users through the application's features. **2** Shiny-Calorie is subdivided into a left sidebar panel for controls, middle panel for configuration and a main panel on the right for visualization, statistical analysis and explanation of the conducted analyses. **2a** Displays the main view with the selected variable of interest over time and **2b** allows to conduct windowed time analysis, which will be added to the main panel below **5** on the bottom. In the main view there is always the possibility to adjust aesthetics of generated plots by using the `Stylize plot` checkbox. **3** Panel to select a metabolic variable for visualization and analysis, also allows for grouping, filtering, annotation of experimental times and advanced options typically not required in a first analysis. **4** Allows for data curation, e.g. trimming of experimental times and, days and sample selection. Raw data curation is specified during the `Dataset import` in **1** and can be configured via the `Import options` section. **6** Allows to export and download all plots and accumulated data frames of calculated and derived quantities as a single compressed `ZIP` archive. The navigation bar links to documentation and Metadata converter Shiny/R app to assist users to fill out the Metadata Sheet [1].

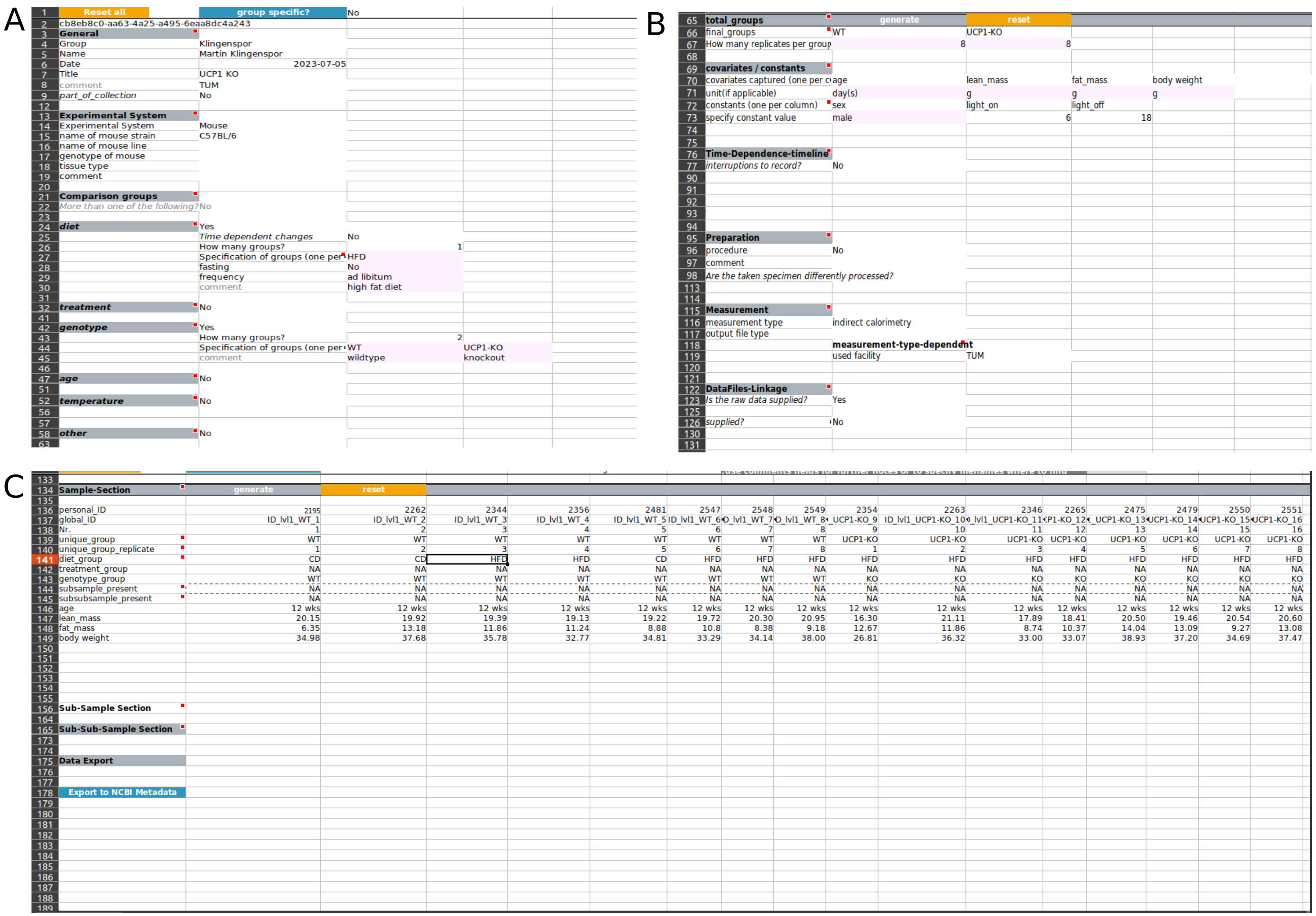

Supplementary Figure S2: **Metadata** as provided through the standardized metadata sheet [1] using a controlled vocabulary provided by the accompanying metadata ontology (not shown). A. Basic description and information of the experiment and experimental setup is given, e.g. diet, treatments, genotype. Other categorical groups can be added *ad libitum* if applicable. B. Specification of measured covariates and constants. C. Compiled sample table for experiment with the possibility for sub-sampling.

	A	B	C	D	E	F	G	H	I	J	K	L	M
1	20200514_SD_Ucpdd_K2		TX001	TX002	TX003								
2		TSE LabMaster V6.3.3 (2017-3514)											
3	Box	Animal No.	Weight [g]	Diet	Genotype	Text3							
4	4	2344	35.78	HFD	WT								
5	5	2346	31.03	HFD	KO								
6	7	2354	33.07	HFD	KO								
7	8	2356	32.77	HFD	WT								
8													
9	Date	Time	Animal No.	Box	S.Flow	Ref.O2	Temp	O2	dO2	dCO2	VO2(3)	VCO2(3)	RER
10					[l/min]	[%]	[°C]	[%]	[%]	[%]	[ml/h]	[ml/h]	
11	14.05.2020	16:30	2344	4	0,4	20,75	25,8	20,32	0,4297	0,3708	135	112	833
12	14.05.2020	16:35	2344	4	0,4	20,74	26,8	20,39	0,3487	0,3011	109	91	834
13	14.05.2020	16:40	2344	4	0,4	20,74	27,5	20,43	0,3094	0,2608	98	79	0,81
14	14.05.2020	16:45	2344	4	0,4	20,75	28	20,44	0,3082	0,2618	96	78	817
15	14.05.2020	16:50	2344	4	0,4	20,75	28,4	20,45	0,2947	0,2539	92	76	832
16	14.05.2020	16:55	2344	4	0,4	20,75	28,7	20,45	0,3057	0,2569	96	77	806
17	14.05.2020	17:00	2344	4	0,4	20,75	29	20,46	0,2885	0,2397	90	72	795
18	14.05.2020	17:05	2344	4	0,4	20,75	29,2	20,47	0,2787	0,2298	86	68	788
19	14.05.2020	17:10	2344	4	0,4	20,75	29,2	20,5	0,2505	0,2112	78	63	0,81
20	14.05.2020	17:15	2344	4	0,4	20,76	29,1	20,52	0,2394	0,2028	75	61	815
21	14.05.2020	17:20	2344	4	0,4	20,75	29,1	20,48	0,2775	0,2446	86	74	855
22	14.05.2020	17:25	2344	4	0,4	20,76	29,2	20,44	0,3192	0,2711	103	84	817
23	14.05.2020	17:30	2344	4	0,4	20,75	29,2	20,56	0,1964	0,1596	62	48	774
24	14.05.2020	17:35	2344	4	0,4	20,76	29,2	20,57	0,1866	0,1532	58	46	784
25	14.05.2020	17:40	2344	4	0,4	20,76	29,3	20,59	0,1682	0,14	53	42	797
26	14.05.2020	17:45	2344	4	0,4	20,76	29,3	20,59	0,1694	0,1439	53	43	817
27	14.05.2020	17:50	2344	4	0,4	20,76	29,4	20,58	0,1805	0,1586	56	48	852
28	14.05.2020	17:55	2344	4	0,4	20,76	29,5	20,44	0,3204	0,2853	99	86	866
29	14.05.2020	18:00	2344	4	0,4	20,76	29,7	20,49	0,2689	0,2362	84	71	851
30	14.05.2020	18:05	2344	4	0,4	20,76	29,7	20,49	0,2738	0,2392	84	71	846
31	14.05.2020	18:10	2344	4	0,4	20,76	29,8	20,43	0,3364	0,2942	104	88	847

Supplementary Figure S3: **Raw data** export as generated by users from a TSE LabMaster v7/v8 system as `CSV` file (viewed in LibreOffice). Metadata is encoded in header rows (1-8) and separated from raw data by a single empty line. Note that raw data is recorded in a row-oriented format for individual samples (e.g. animals) here, raw data can also be exported into a column-oriented format (not shown).

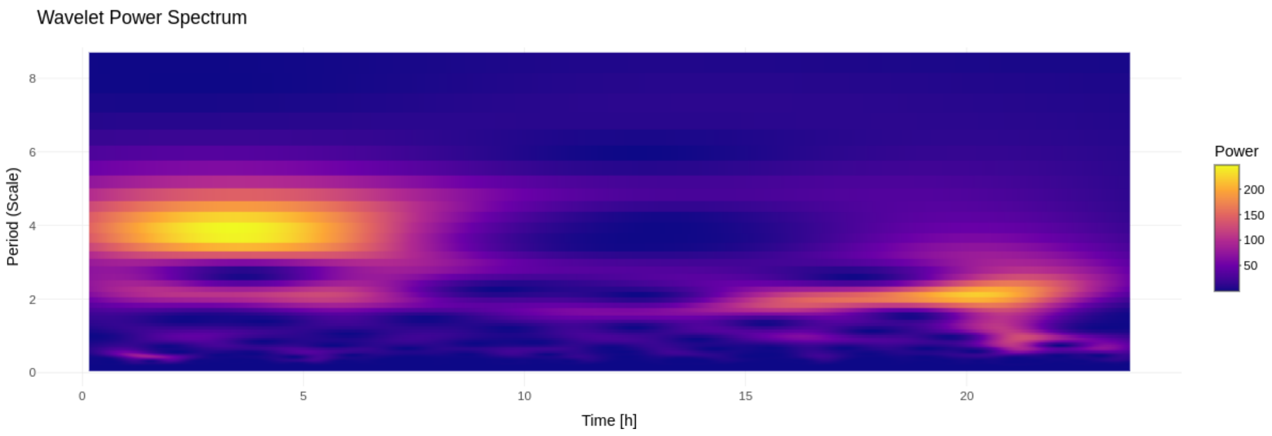

Supplementary Figure S4: **Wavelet power spectrum.** A detailed visualization of the dominant frequency components with changes over time is shown for the signal of metabolic rate of a single individual sample.

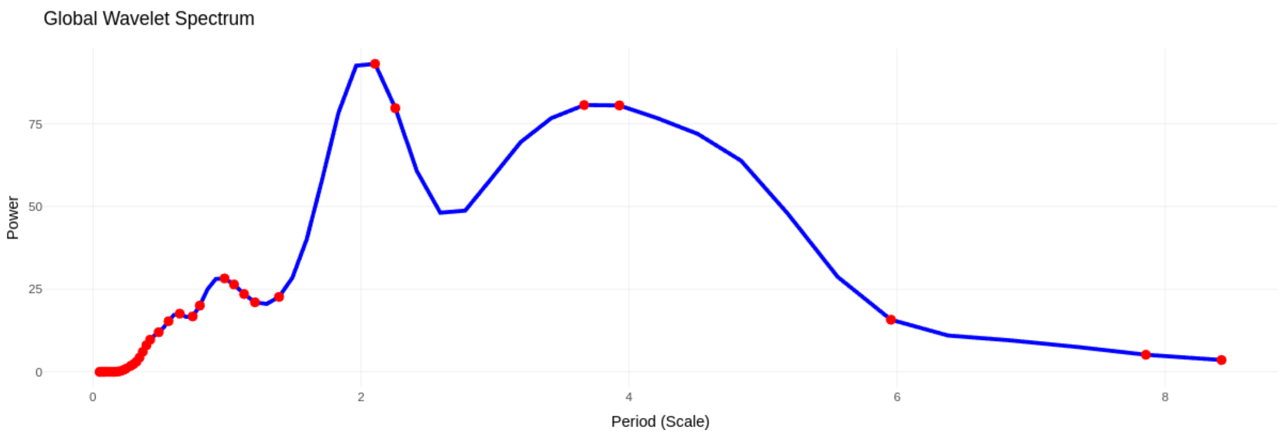

Supplementary Figure S5: **Significant periods in the global wavelet spectrum.** Significant frequency components are visualized which are present in the analyzed non-stationary signal of metabolic rate for a single individual sample.

Supplementary Table S1: **Supported input file formats**. Tabulated are all currently, i.e. in Shiny-Calorie v0.4.9 version, supported file formats as output from the associated metabolic phenotyping platforms. File types are allowed to be combined during upload (multiple cohort study) and their metadata labels are harmonized.

**Manufacturer**	**File type**	**Supported version**	**Format specification**
Sable Systems	.xlsx	$\geq$ v22.0.0	Promethion Live
TSE Systems	.csv / .tsv	$\geq$ v5.0.0, $\geq$ v6.0.0	LabMaster
TSE Systems	.csv / .tsv	$\geq$ v7.0.0, $\geq$ v8.0.0	PhenoMaster
PhenoSys	.csv	-	Calobox
COSMED Generic	.xlsx	-	-
COSMED QNRG	.csv	-	-
Columbus Instruments	.csv	-	CLAMS Oxymax
CaloPy	.csv	v1.0.0	-
CalR	.csv	v2	-

Supplementary Table S2: **Available heat production equations**. Tabulated are the available heat production equations with definition, units and literature reference. For a discussion of these equations, i.e. how the equations have been derived from experimental data sets, their application domains and physiological underpinnings and implications, cf. [2]. Note that the factor $\frac{3.6}{1000}$ in the Heldmaier equations converts mW to $\frac{kJ}{h}$ for compatible output units for energy expenditure calculations.

**Name**	**Equation**	**Input**	**Output**	**Reference**
Heldmaier #1	$(4.44 + 1.43 \times RER) \times \dot{V}O_2 \times \frac{3.6}{1000}$	$\frac{ml}{h}$	$\frac{kJ}{h}$	[3]
Weir	$0.0163 \times \dot{V}O_2 + 0.0457 \times \dot{V}CO_2$	$\frac{ml}{h}$	$\frac{kJ}{h}$	[4]
Ferrannini	$0.01637117 \times \dot{V}O_2 + 0.0046057 \times \dot{V}CO_2$	$\frac{ml}{h}$	$\frac{kJ}{h}$	[5]
Lusk	$0.01579 \times \dot{V}O_2 + 0.00509 \times \times \dot{V}CO_2$	$\frac{ml}{h}$	$\frac{kJ}{h}$	[6]
Elia	$0.0158 \times \dot{V}O_2 + 0.00518 \times \dot{V}CO_2$	$\frac{ml}{h}$	$\frac{kJ}{h}$	[7]
Brouwer	$0.01607 \times \dot{V}O_2 + 0.00469 \times \dot{V}CO_2$	$\frac{ml}{h}$	$\frac{kJ}{h}$	[8]

Supplementary material for Shiny-Calorie: A context-aware application for indirect calorimetry data analysis and visualization using R

Stephan Grein[1] Tabea Elschner[2,3,†] Ronja Kardinal[4,†] Johanna Bruder[5] Akim Stromeyer[5] Karthikeyan Gunasekaran[6] Jennifer Witt[6] Hildigunnur Hermannsdottir[7] Janina Behrens[6] Muuez U-Din[8] Jiangyan Yu[9] Gerhard Heldmaier[10] Renate Schreiber[11] Jan Rozman[12] Markus Heine[6] Ludger Scheja[6] Anna Worthmann[6] Joerg Heeren[6] Dagmar Wachten[4] Kerstin Wilhelm-Jüngling[2,3] Alexander Pfeifer[13] Jan Hasenauer[1,*] Martin Klingenspor[5,7,14,*]

1 Life and Medical Sciences (LIMES) Institute and Bonn Center for Mathematical Life Sciences, University of Bonn, Bonn, Germany
2 Institute for Cardiovascular Sciences, University Hospital, University of Bonn, Bonn, Germany
3 Institute for Neurovascular Cell Biology, University Hospital, University of Bonn, Bonn, Germany
4 Institute of Innate Immunity, University Hospital, University of Bonn, Bonn, Germany
5 EKFZ - Else Kröner-Fresenius Center for Nutritional Medicine, Technical University of Munich, Freising-Weihenstephan, Germany
6 Department of Biochemistry and Molecular Cell Biology, University Medical Center Hamburg-Eppendorf, Hamburg, Germany
7 Chair of Molecular Nutritional Medicine, TUM School of Life Sciences, Technical University of Munich, Freising, Germany
8 Turku PET Centre, University of Turku and Turku University Hospital, Turku, Finland
9 Institute of Clinical Genetics and Genomic Medicine, University Hospital, Würzburg, Germany
10 Animal Physiology, Department of Biology, Marburg University, Marburg, Germany
11 Institute of Molecular Biosciences, University of Graz, Graz, Austria
12 Luxembourg Centre for Systems Biomedicine, University of Luxembourg, Esch-Belval, Luxembourg
13 Institute of Pharmacology and Toxicology, University Hospital, University of Bonn, Bonn, Germany
14 ZIEL - Institute for Food & Health, Technical University of Munich, Freising, Germany

1 Introducing Shiny-Calorie

Shiny-Calorie, a context-ware application for indirect calorimetry data analysis and visualization using R, is an open-source, reactive web application for the comprehensive visualization and statistical analysis of general indirect calorimetric data sets arising from indirect calorimetry experiments using common metabolic phenotyping platforms, i.e. TSE Systems Phenomaster/LabMaster, Sable Systems Promethion and the COSMED platform, cf. Tab. S1. The previously referenced table contains a list of supported file formats and versions for the mentioned metabolic phenotyping platforms. Shiny-Calorie ingests data and standardized metadata, and automatically integrates and harmonizes the data sets for analysis. For an overview of Shiny-Calorie's user interface, cf. Fig. S1.

Usage of the web application The Shiny-Calorie web application is deployed on our Kubernetes cluster `https://shiny.iaas.uni-bonn.de/Shiny-Calorie` with built-in redundancy for high availability.

Common web browsers in recent versions are suggested (e.g. Chrome/Chromium, Firefox), since Shiny/R inherently makes use of the web technology stack, e.g. CSS, JS, etc., for rendering of content and user interaction.

Users interface through the browser to load, analyze data and generate visualizations of indirect calorimetry experiments and conduct statistical hypothesis testing. Two published ready-to-use example datasets (UCP1-KO and ADKO study) are integrated into the web application (datasets are loadable via two different buttons indicating the dataset directly on the application's landing page) for a quick exploration of the functionality of Shiny-Calorie. The same datasets are detailed and analyzed in Shiny-Calorie's accompanying documentation on `https://ICB-DCM.github.io/Shiny-Calorie/`. Additionally, users can compile their custom datasets by use of our calorimetry tools (using the Apache/Solr2 REST API) accessing the International Mouse Phenotyping (IMPC) database, see Section 6.3 on IMPC dataset generation.

Nota bene: To support efficiently multiple users in their analysis requirements, we utilize a custom lab-internal small-scale Kubernetes cluster (4 worker nodes and 1 master controller node). This allows us to load balance and upscale on demand to support efficient usage of the web application and adapt to growing number of users if necessary.

2 On-premise installation and deployment

The Shiny-Calorie web application is made available through a containerized OCI docker image via `hub.docker.com`, cf. repository `https://hub.docker.com/r/stephanmg/Shiny-Calorie` for details. Docker images can be accessed via the common workflow, i.e. `docker pull stephanmg/Shiny-Calorie` to obtain the latest tagged version.

Typically Shiny-Calorie will be placed behind a reverse-proxy solution for load balancing with e.g. Nginx on local compute infrastructure (to allow for efficient parallel usage by many different users) or in a setup involving Kubernetes. Start scripts are provided in the source code repository on Github, and an example configuration to set up load balancing via Nginx for multiple parallel running app instances to support efficient multi-user usage of the web application is provided. Thus, Shiny-Calorie can be installed easily on either local computers as well as on cloud infrastructures respectively IaaS providers by means of Docker or drop-in replacement as Podman or the analogues Singularity/Apptainer.

3 Installation on a personal computer

For Shiny-Calorie builds are created nightly, i.e. installers for Windows, OSX and Linux (Ubuntu) are available as artifacts from the Github Actions workflows on Shiny-Calorie's Github repository. Standalone versions are provided for situations where connectivity is limited, i.e. in offline field experiments or shielded lab rooms with low or no connection strength for wireless transmission where also no Ethernet is available.

4 Code availability

The code of Shiny-Calorie is available in the corresponding Github repository, and installation from source is possible with dependencies managed through `renv`, rendering a native installation of Shiny-Calorie convenient for developers and promotes reproducibility. The corresponding `renv.lock` file is contained within the repository itself, listing required packages and dependencies.

5 Documentation

The application provides built-in documentation, see top right corner in the web application. A walk-through, respectively, a guide is available at the user's discretion ('Guide' button in web application) on the web application's landing page. Further documentation is available on the RTD documentation of Shiny-Calorie.

6 Application examples

In the following sections, two application examples illustrate the common workflows of analysis for indirect calorimetry experiments with example datasets. The examples are intended to guide new users and to become familiar with the features, layout of the user interface and the functionality of Shiny-Calorie. These examples were developed in close collaboration with wet-lab scientists and are based on routinely applied standard operating procedures (SOPs) and analysis protocols in the labs. The methods implemented in Shiny-Calorie automatize repetitive day-to-day workflows by a joint integration of data and metadata as recorded in the lab book using the Metadata sheet.

Additional guidance and documentation are available through the built-in help system and step-by-step tutorial embedded within the application's user interface. Furthermore, two additional use-cases respectively examples are described in tutorial style on Shiny-Calorie's documentation website: `https://ICB-DCM.github.io/Shiny-Calorie`

6.1 Dataset I: Disentangle activity-related energy expenditure from resting metabolic rate and statistical hypothesis testing on data stratifications

To illustrate the workflow of data analysis after an indirect calorimetry experiment has been conducted, we consider the case of reconstructing energy expenditure (EE) and analysis thereof and perform additional subsequent downstream analysis tasks, i.e. calculation of the resting metabolic rate (RMR), total energy expenditure (TEE) and the activity-related energy expenditure (AEE) stratified by genotype. We consider here a stratification with respect to the genotype, which is a specific choice made to illustrate the dataset. It would also be possible to group by diet, condition, temperature and related variables.

Prior to initiating any analysis in Shiny-Calorie, the raw input data—acquired from one of the supported

metabolic phenotyping platforms (MPPs)—must successfully pass the built-in consistency checks. Shiny-Calorie enables the analysis of indirect calorimetry datasets from multiple experiments or cohorts, which can be stratified by variables such as genotype or treatment condition (e.g., cold exposure), or analyzed jointly in multi-factorial designs. By default, metadata are extracted from the headers of commonly supported file formats. For enhanced standardization and richer annotation, users are encouraged to supply a structured Metadata Sheet [1].

In the following, we outline the individual steps:

1. **Dataset import:** The user-provided indirect calorimetry datasets are imported into the application using the upload dialog. The supported file types and formats are listed in Tab. S1. Labels of different datasets are harmonized automatically without user intervention. The application outputs a warning if not fully-supported datasets are provided and restricts the users from further analysis unless explicitly ignored by the user.

File upload dialog for data sets:

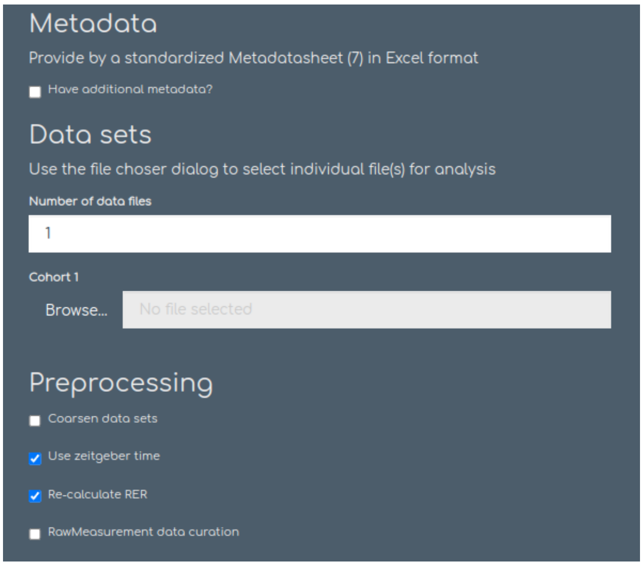

During data import, the user can either flag or remove implausible values through invoking the raw data curation panel. Pre-processing of raw datasets is thus either automatically handled or flagged for manual inspection by users. Pre-processing allows to coarsen the dataset (i.e. short measurement intervals), interpolate to a regular time grid (if unequal measurement interval lengths are given), convert to zeitgeber time (align with the photoperiod) or keep regular calender time. We allow to recalculate the respiratory exchange ratio too, as we observed that some platforms do not correctly output the quantity in `CSV` files occasionally.

Checking the box `RawMeasurement data curation` displays additional panels which various options for outlier removal and marking of outliers, and enables the possibility to activate the raw data consistency checks.

Pre-processing panel:

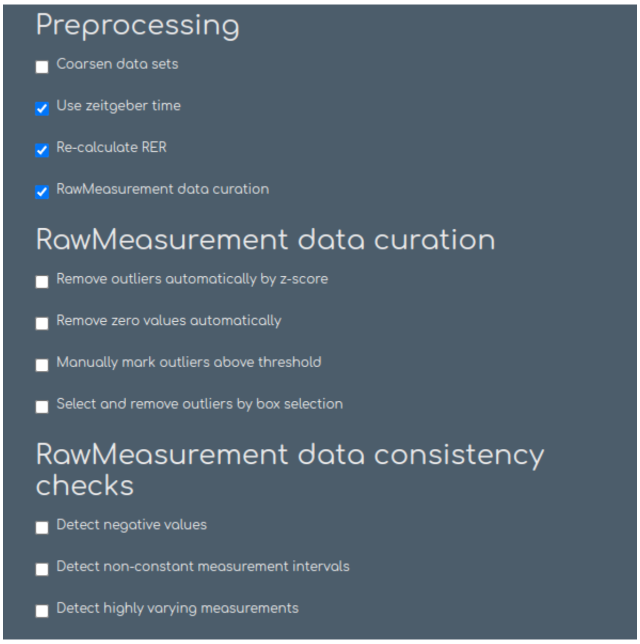

2 **Review of metadata:** The users review the metadata attached to their indirect calorimetry experiments. Purposefully Shiny-Calorie outputs a comprehensive overview of metadata to allow for a detailed analysis of metadata, numerical quantities are output as density plots and categorical data as boxplots. Users compare all metadata and perform multi-way ANOVAs in the `Statistical testing` panel to check consistency of experimental design (and for biases), e.g. even distribution of diets or genotypes, consistent age of samples, or to check for any significant differences in, e.g., body weight which might distort statistical analysis when correcting for body weight to avoid biased analyses.

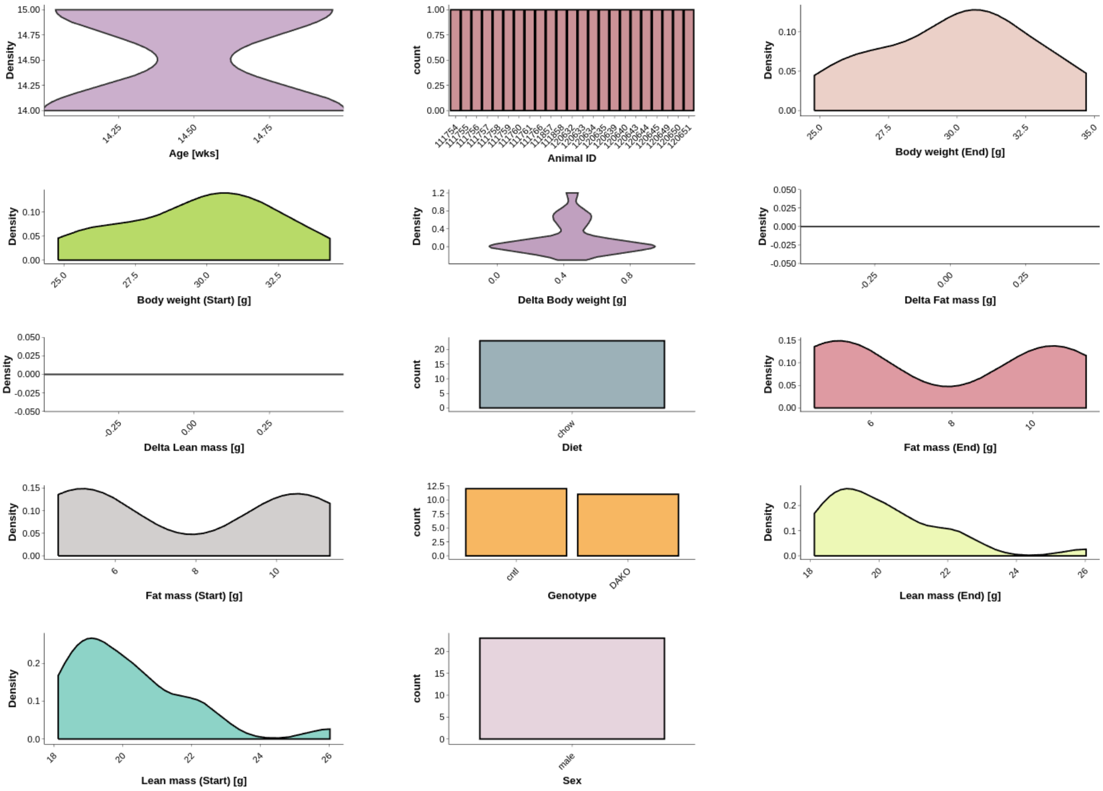

Following initial data visualization and statistical testing, users should ensure that no major biases exist in the experimental design before proceeding with additional quality control steps. This includes the routine inspection of respiratory gas exchange traces (CO_2 and C_2) and the respiratory exchange ratio (RER), which serve as critical indicators of measurement integrity in indirect calorimetry experiments which is calculated as the fraction of the former quantities.

The metadata review acts as an initial checkpoint to confirm consistency and completeness of the experimental setup. Raw data inspection involves not only assessing the gas exchange profiles and RER, but also, where available, evaluating additional parameters such as temperature and photoperiod information. These supplementary variables help identify inconsistencies or anomalies in the exported data from the metabolic phenotyping platforms.

Statistical testing for categorical metadata:

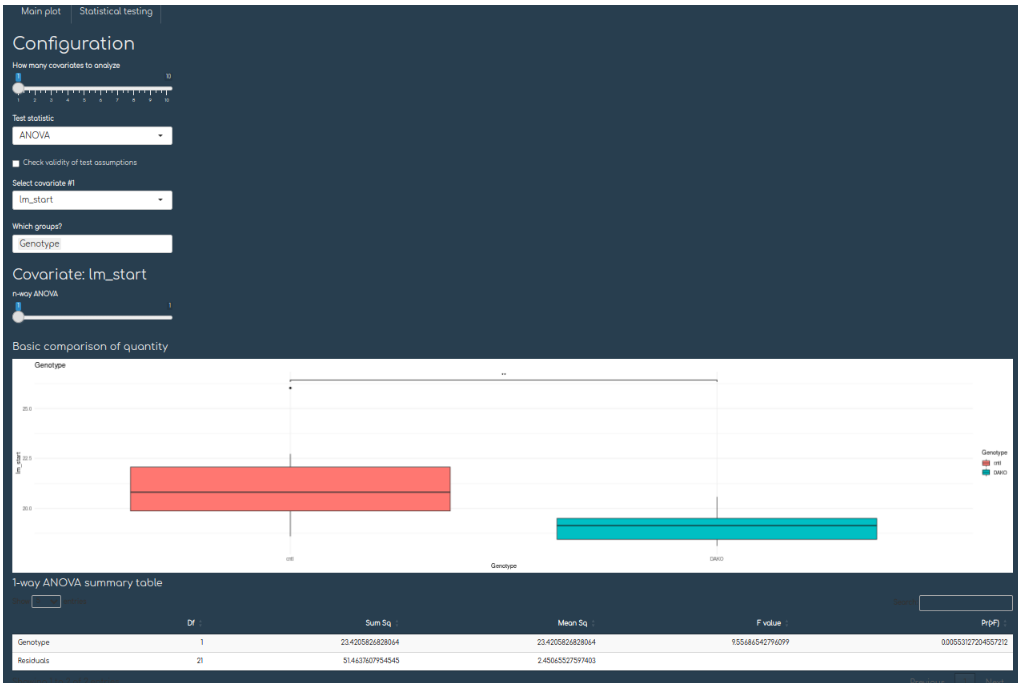

The consistency of input datasets is vital, and thus should always be verified prior to any calculations of EE, analysis, or statistical hypothesis testing thereof. The first important step is concerned with the inspection of the raw oxygen and carbon dioxide signals (saturation and actual production and consumption), as well as derived quantity i.e. the respiratory exchange ratio (RER) mentioned earlier. These quantities should be examined for inconsistencies or irregularities.

The respiratory exchange ratio (dimensionless) derived measurement visualized over time course of experiment can give important information about substrate utilization and soundness of the indirect calorimetry experiment:

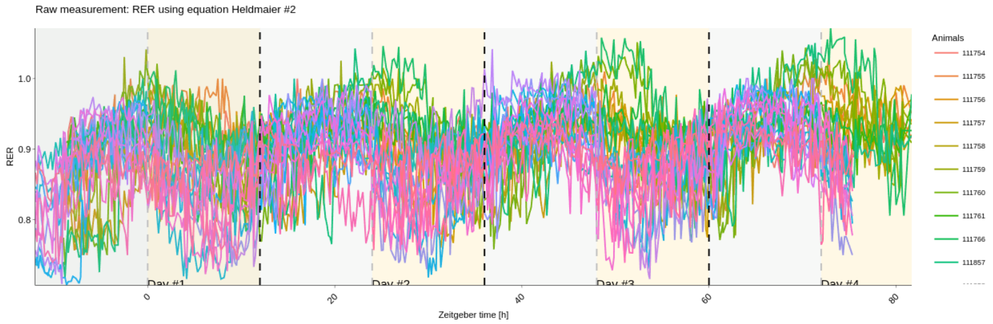

Furthermore, the inspection of the raw oxygen and carbon dioxide signals can help detect experimental flaws or issues with subjects in the calorimetric cage or chamber.

Users can request that subsequent analysis should be prohibited when high-frequency (non-physiologically) measurements or non-plausible input data (e.g. negative values) are detected, resulting in a visual pop-up warning in the web application with additional information in the `Data set loading` panel, cf. Fig. S1 section 1. The warning can be overridden at the discretion of the user.

Plot of oxygen consumption (VO_2) as a raw measurement visualized over time course of experiment:

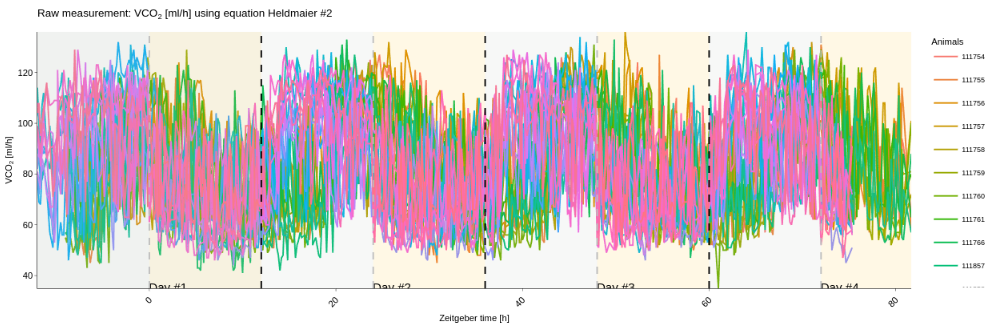

Plot of carbon dioxide production (VCO_2) as a raw measurement visualized over time course of experiment.

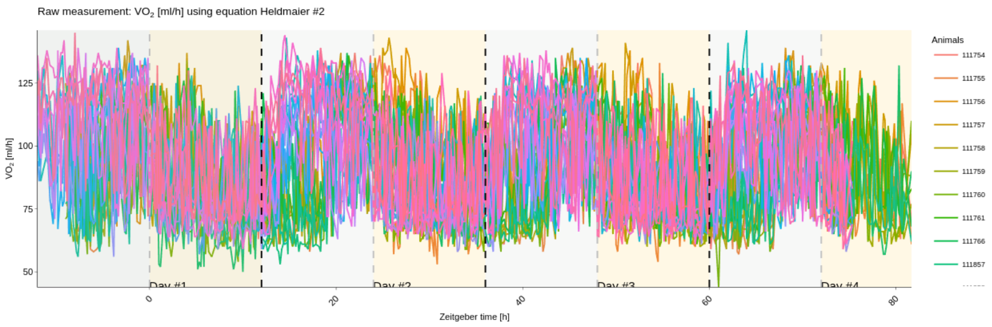

Additionally, the signals of O_2 and CO_2 saturation should be inspected. A visualization of these quantities can reveal if there are any general issue with the indirect calorimetry experiment, i.e. air flows within the metabolic cages, or an issue with the MPP as set up in the laboratory environment. Empty control cages can be excluded from analysis as well.

Plot of the raw measurement oxygen saturation:

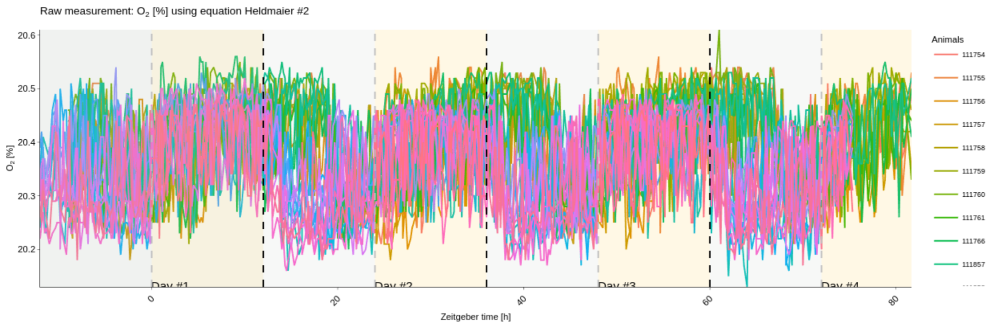

Plot of the raw measurement carbon dioxide saturation:

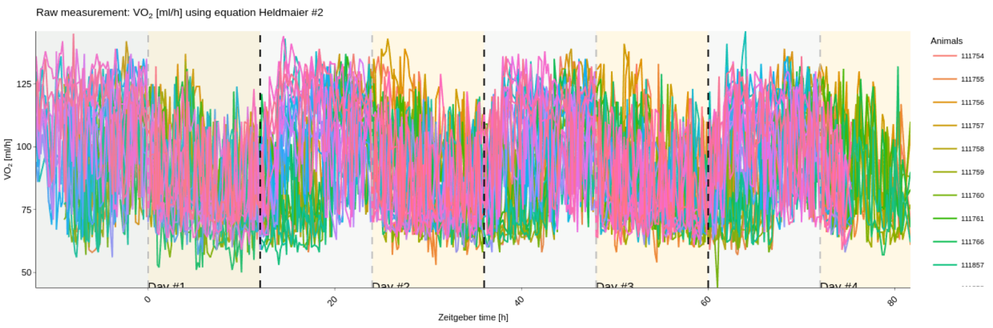

3 **Data curation:** If inconsistencies or irregularities in datasets have been detected, the user can request the removal of these outliers (e.g. exclude sick or erratic animals), trim considered experimental times from the start or end of the indirect calorimetry experiments or select a subset of given experimental day only. Trimming of data might be necessary because of the time-dependent accommodation and acclimatization of animals in metabolic cages. Opening the cage or handling the laboratory animals, distorts the O_2 or CO_2 signal (or derived quantities i.e. RER) in the early and late stages of an experiment.

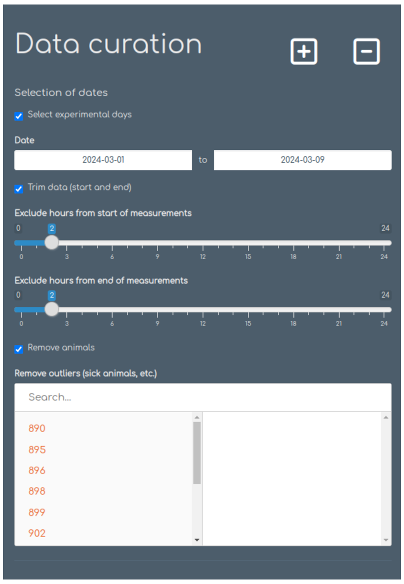

Without correcting for experimental flaws or implausible values, subsequent downstream analysis will receive eventually inconsistent input data and results and statistical analysis are biased. Date ranges are specified via the data input ranges for start and end of experiment. Hours at the beginning or end of an experiment can be trimmed by utilizing the provided sliders to trim from start and end respectively. Sick or erratic animals can be excluded by selecting animal identifiers (sample IDs) in the selection menu. Note data curation is available for all visualizable quantities which users can select in Shiny-Calorie.

4 **Reconstruction of energy expenditure**. The user selects a heat production equation for their experiments. Commonly used heat production formulas to reconstruct energy expenditure and derived quantities are available in Shiny-Calorie (Tab. S2). By a modular design additional heat production formulas can easily be integrated by R users. For exploratory testing, the user can chose two heat production formulas and test for differences in heat production based on the choice they made in an energy expenditure heat production formulas scatter plot. The chosen heat production formula will be used during all downstream analysis and visualization tasks and can be overridden with this single configuration option to allow for a consistent and transparent analysis. In general quantities can be grouped or filtered by metadata, not only for energy expenditure but other quantities.

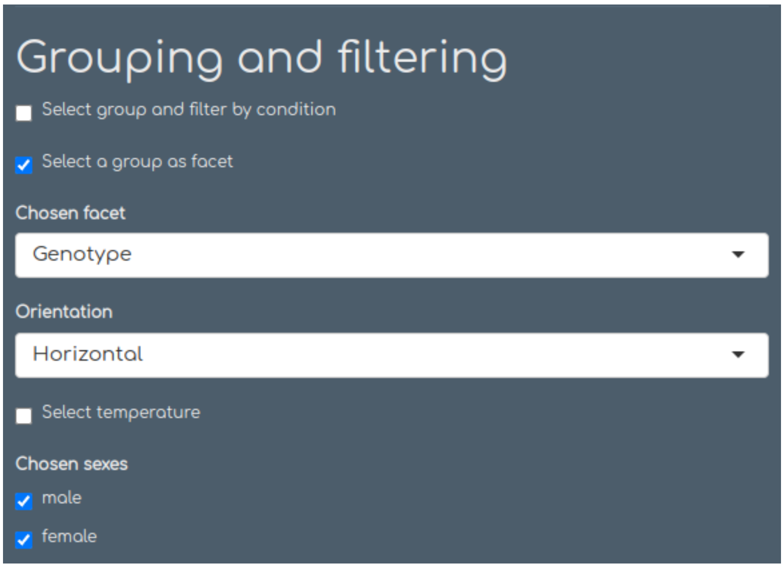

Grouping and filtering. Any raw, calculated or derived quantity can either be filtered by using a level or grouped by facets (factors) based on imported metadata. Further samples can be filtered for temperature and based on sex.

Nota bene: The choice of a specific heat production formulas had empirically neither a pronounced nor significant difference on energy expenditure as calculated from our test datasets using the raw curated or non-curated measurements from the indirect calorimetry experiment. The user can determine the effects of different heat production equations for energy expenditure calculation using their dataset by using the plotting routine `CompareHeatProductionFormulas` from the `Variable selection` section by selecting the`General` indirect calorimetry platform.

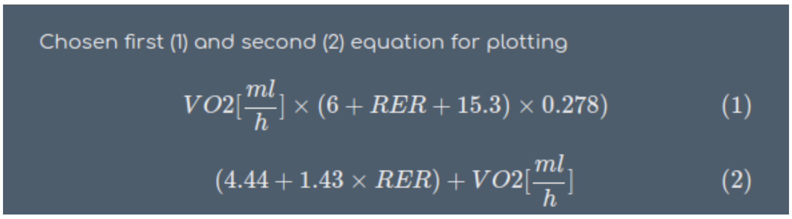

Heat production equation. Choosing from the available heat production equations for energy expenditure calculation from respiratory gas exchange data, see also Tab. S2 for options.

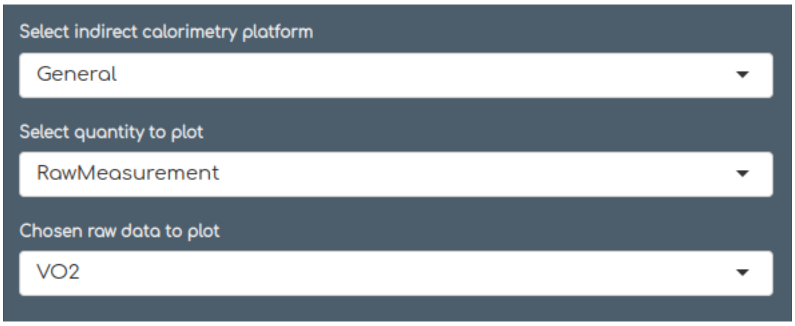

Selection of analysis. Choose a raw quantity, derived quantity or energy expenditure quantities to visualize.

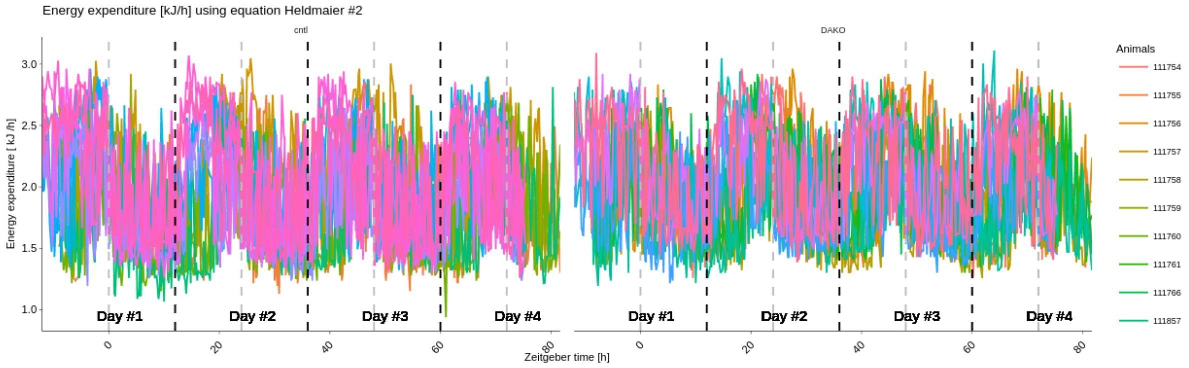

Energy expenditure over time stratified by the condition diet. Individual energy expenditure traces of animals are shown and can interactive be controlled in plots with the Plotly library.

5 **Check point.** After all of the previous steps do not detect irregularity or inconsistencies, neither visually nor through statistically testing, users can proceed with downstream analysis.

6 **Calculation of derived quantities**. Resting metabolic rate, total energy expenditure and activity-dependent energy expenditure can be visualized as well.

RMR stratified by genotype for each individual subject:

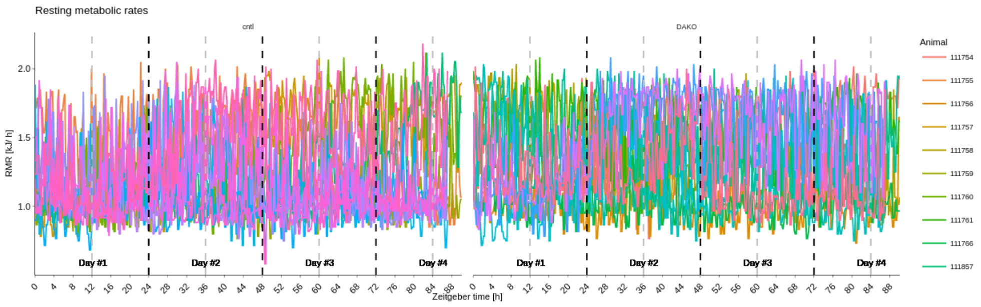

TEE stratified by genotype for each individual subject:

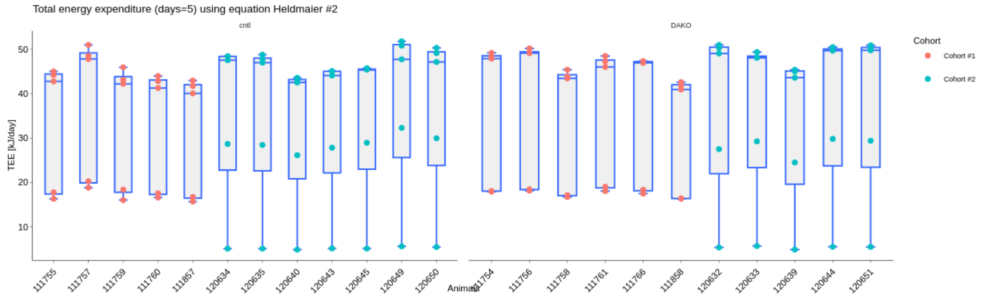

Separation of AEE and RMR:

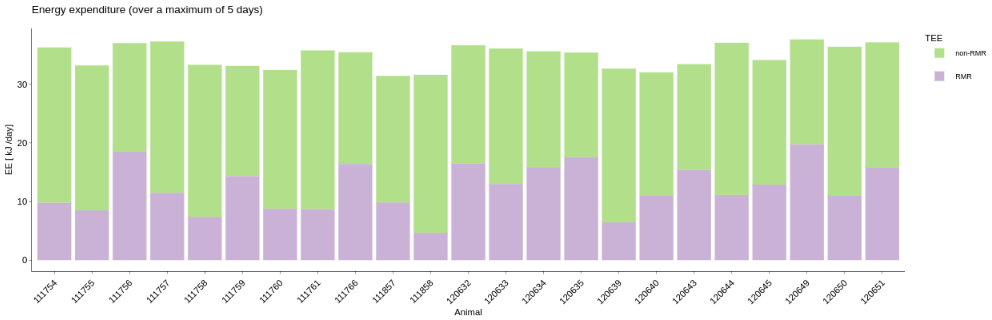

Shiny-Calorie allows also for trend detection, by estimation of the mean traces for all metabolic variables (using GAMs). Mean traces for RMR stratified by genotype:

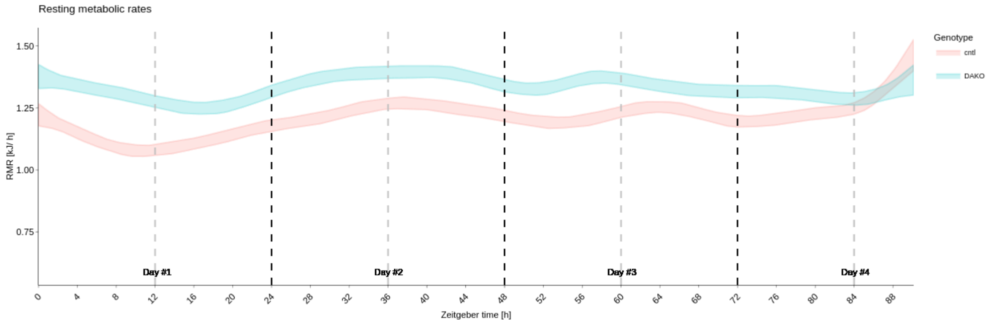

7 **Statistical analysis:** A typical analysis case in indirect calorimetry experiments is concerned with a discrimination of genotype or diet effect on RMR and TEE (and which amount is related to AEE), i.e. energy expenditure stratified across conditions (genotype, diet or both) is of interest to the researchers. In this application example, we compare genotypes (wildtype (WT) and knockout (KO)) with identical diets for an all-male cohorts experiments recorded with the TSE metabolic phenotyping platform.

Thus, the researcher is left with the following steps:

- Calculate total energy expenditure: TEE
- Calculate resting metabolic rate: RMR
- Contrasting and statistical testing of TEE, AEE and RMR

After calculation of TEE and RMR (also termed non-activity related energy expenditure) the following hypothesis should be answered: Is there a genotype effect in RMR or TEE depending on the WT or KO genotype for these cohorts. To answer these questions, the user can conduct a 1-way ANCOVA or 2-way ANCOVA. When a second independent grouping variable, e.g. diet information, becomes or is available through the provided metadata sheet, multi-factorial designs are available to the users. For a visualization of TEE (or EE) and RMR quantities as boxplot or scatte rplots with regression lines, see below.

Boxplot comparison of TEE stratified by genotype, p-values are reported based on ANOVA with Wilcoxon-test.

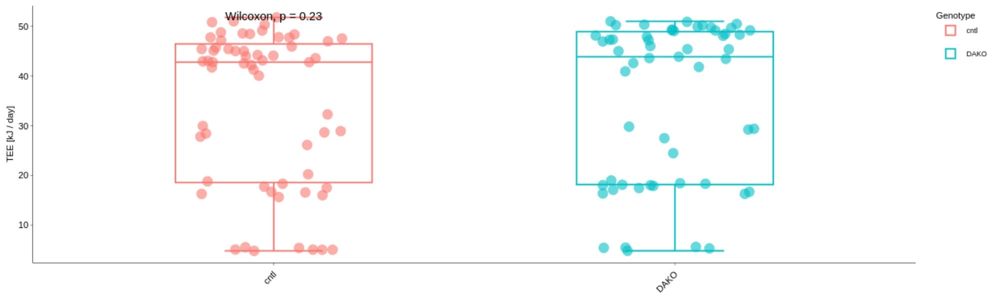

A scatter plot with regression lines for each genotype, R^2-values are reported:

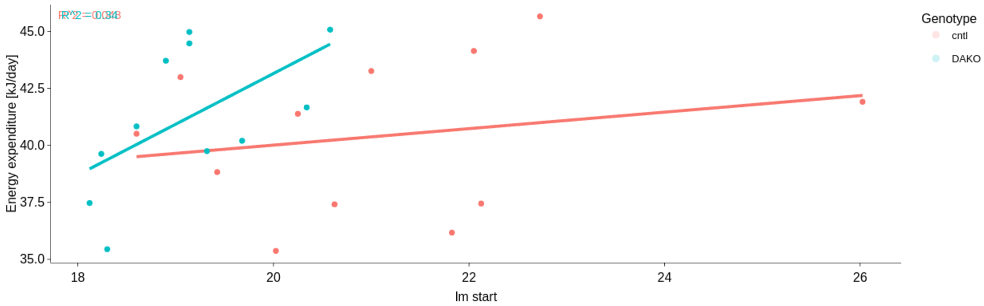

Prior to outputting statistics and conducting of statistical testing, test assumptions, e.g. normality of residuals homoscedasticity of data are verified through visuals, e.g. diagnostic plots, in the application and p-values are reported following. The user can switch between parametric and non-parametric testing depending on the properties of the data set.

When test assumptions and significance levels are met as required by the corresponding tests, conclusions can be drawn and data sets and high-quality figures exported.

1-way ANCOVA comparing KO and WT genotype corrected for body weight variation reveals no difference in RMR and in agreement with previously published results [9].

Test assumption for parametric testing (ANOVA/ANCOVA), e.g. normality of residuals, homoscedasticity and homogeneity of regression slopes are checked and reported to the user.

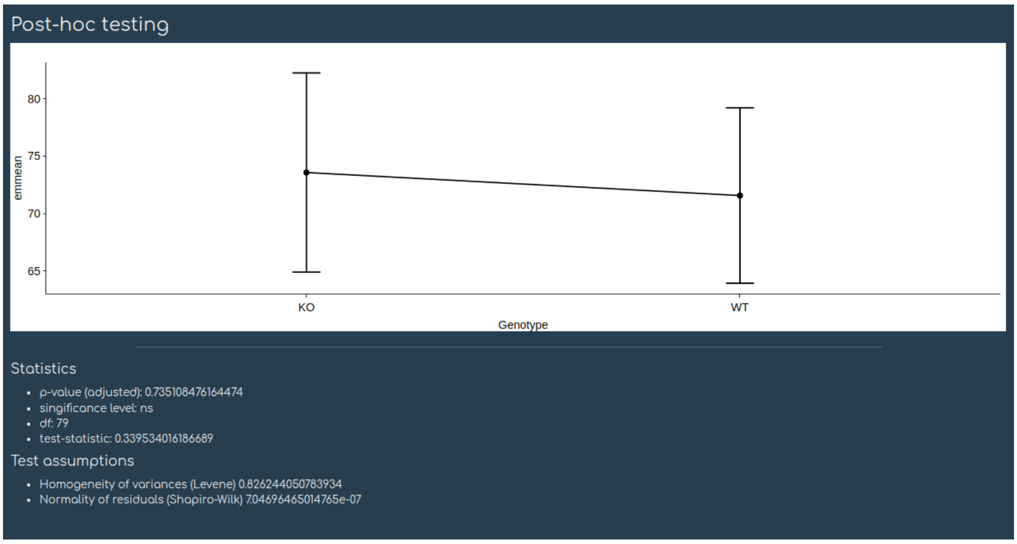

To check for applicability (validate test assumptions for parametric testing) of a statistical test, the corresponding checkbox should be marked:

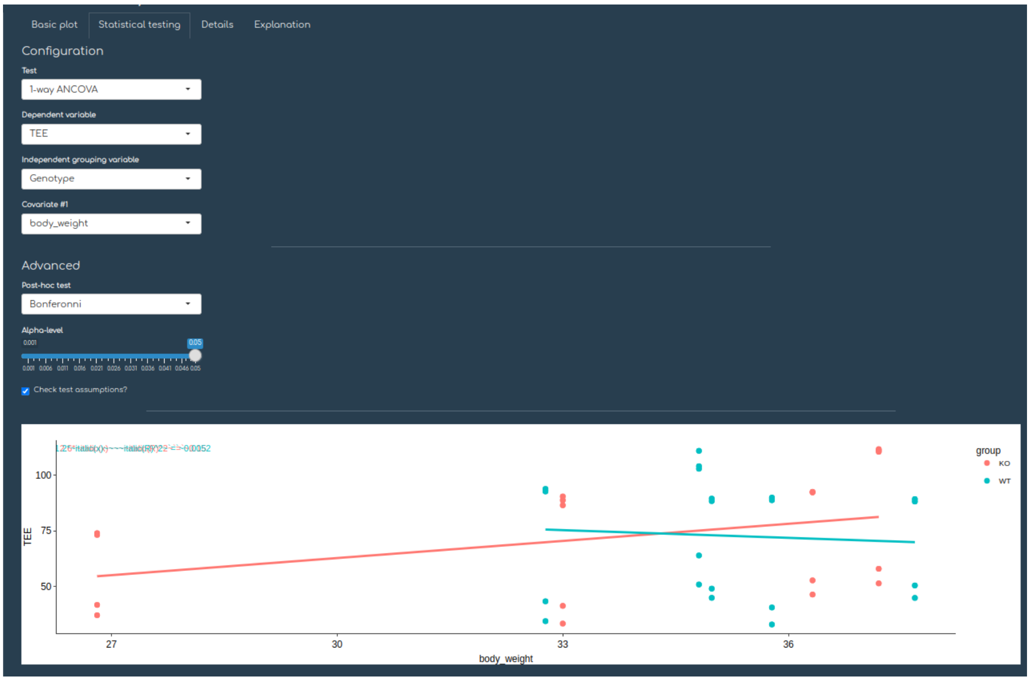

Estimated marginal means for an exemplary 2-way ANCOVA:

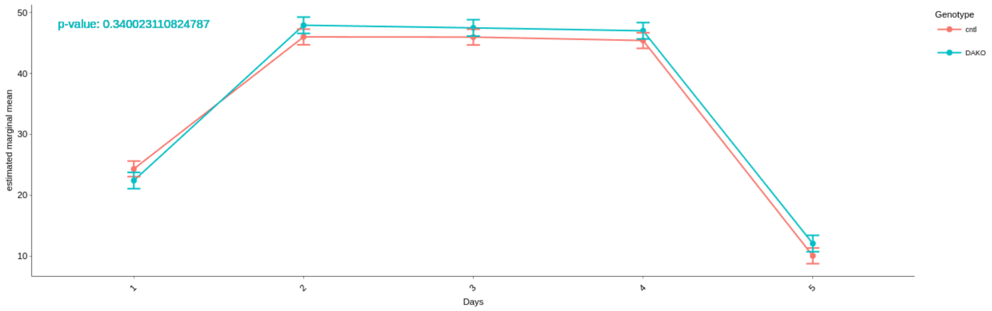

Box plots for the 2-way ANCOVA with additional grouping by Days:

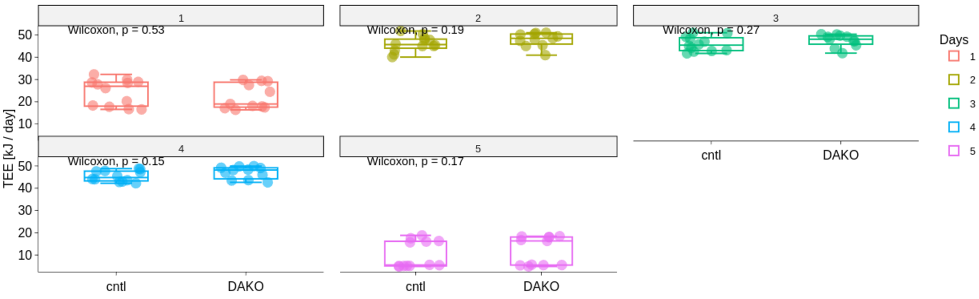

Typically ANCOVA is performed with whole body weight as a covariate we require to correct for, however, by the availability of additional metadata, it is useful to correct for either lean, fat or the combination of lean and fat body mass instead, as is done recently in increasing numbers for metabolic phenotyping experiments. Furthermore TEE and RMR budgets can be visualized, concluding the analysis of TEE and RMR.

In addition windowed time-trace plots can be generated which display per window (e.g. 5, 10, 20 or 30 minutes) averaged quantities of samples. The windowed time-trace plots render useful, as otherwise grand averages might hide significant differences in the course of a day. In the following plot, statistically significant differences are denoted by an asterisk (*).

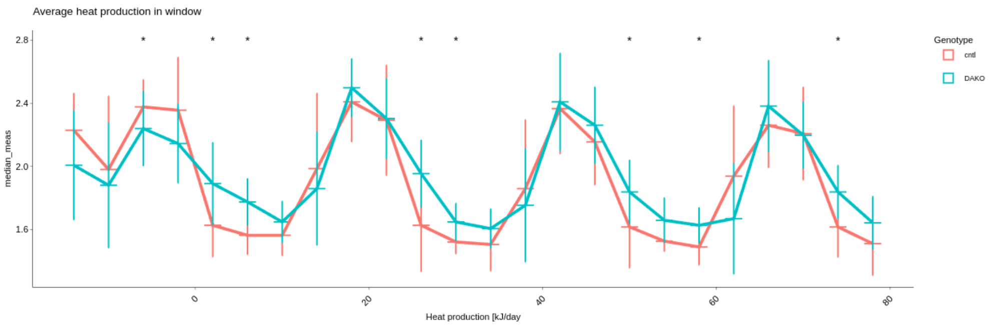

Windowed-time trace analysis reveals, that dependent on the time of day genotype differences can be detected, contrary to analyzing only day-averaged quantities.

6.2 Dataset II. Investigation of locomotion and locomotion budget as a proxy for soundness of experimental setup

It is imperative, whenever locomotion data is available, to verify the behavior of animals in metabolic cages to identify irregularities, idiosyncrasies or problems in experimental setup or with animals, or problems arising during the course of the metabolic phenotyping experiment, which could bias analysis of energy expenditure.

Some metabolic phenotyping platforms, e.g. Sable systems (and also recent versions of TSE Systems. i.e. PhenoMaster), record a subset of locomotion data (steps and time spend in a certain location in the cage by recording time and position (in the (Y, X)-plane using cartesian coordinates) or by recording utilization of the spinning wheel installed in the metabolic cage. A valuable tool therefore is to analyze plots of locomotion density maps (probability density maps), which then can be used to inspect respectively screen for potential issues in indirect calorimetry experiments. Also, the locomotion can be attributed to certain predefined types of physical activity, e.g. drinking, food or using the spinning wheel. A behavioral analysis can be conducted and the locomotion budget can be tabulate and visualized through stacked bar plots. Gained insight can be used for downstream analysis (or in data curation to remove outliers) and the consistent quantification of energy expenditure into the metabolic components activity-related energy expenditure and resting metabolic rate.

Percentage of different locomotional activities (with labels defined as in the Sable/Promethion system) stratified by animal IDs:

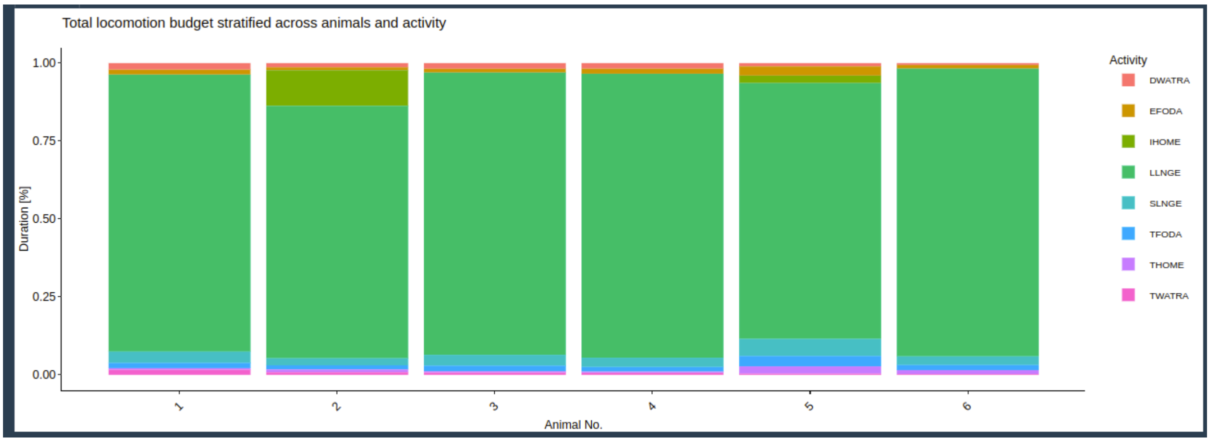

Additionally, not only the locomotor activity can be analyzed but also the budget of locomotion be summarized. In particular a probability density map of animal position in the metabolic cage can be visualized for the purpose of experimental validation. Animal positions are calculated from indirect calorimetry data measuring position of animals at time t in the (Y, X) plane. Dimensions of food hopper (as specified in metadata by Sable/Promethion in Excel workbook), running wheel and water bottle are indicated by colored rectangles.

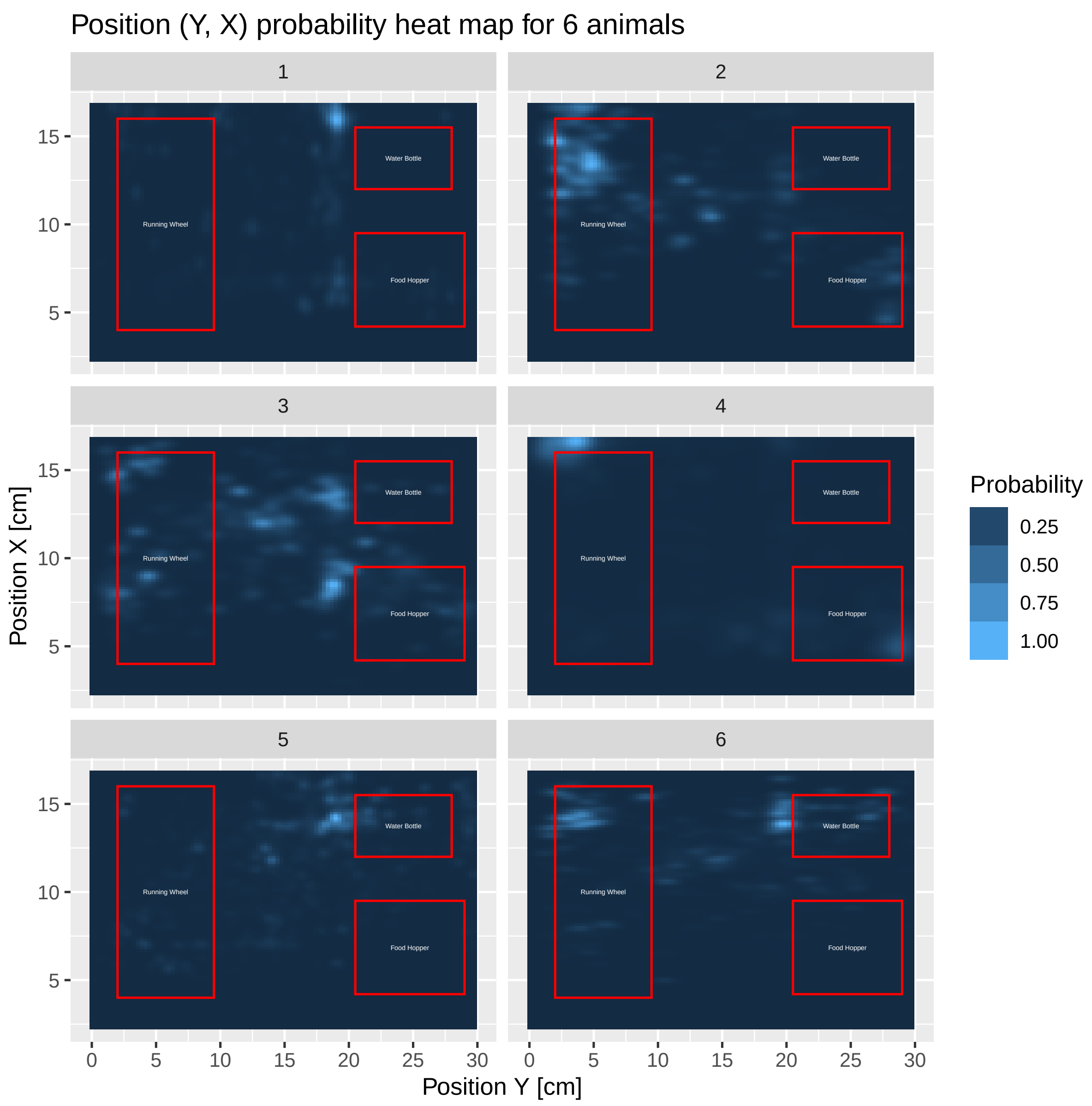

To explore in more depth the dataset and behavior of animals, perhaps to identify issues in experimental setup as mentioned in the previous paragraph, Shiny-Calorie allows for the comprehensive visualization and analysis of related quantities e.g., feeding, drinking, individual step counts, odometer or tracking of body weight. Body weight tracking shows stepwise (discrete) increases and decreases, because animals only sporadically use the scale in the metabolic cage to allow for the measurement of the total body weight.

Step counts are discrete and determined by animals walking through laser light barriers, similarly the distance traveled is determine by taking into account the time between two events of breaking the barrier.

Additionally some setups use a spinning wheel to allow for physical activity of animals, which is measured as another covariate (discrete step counts). Drink and food can be monitored quasi continuously from the provided food in the hamper and water in the drinking bottle. Similarly feeding and drinking periods can be summarized to use the information stored in the IC dataset comprehensively.

Cumulative discrete count of steps in (Y, X) plane:

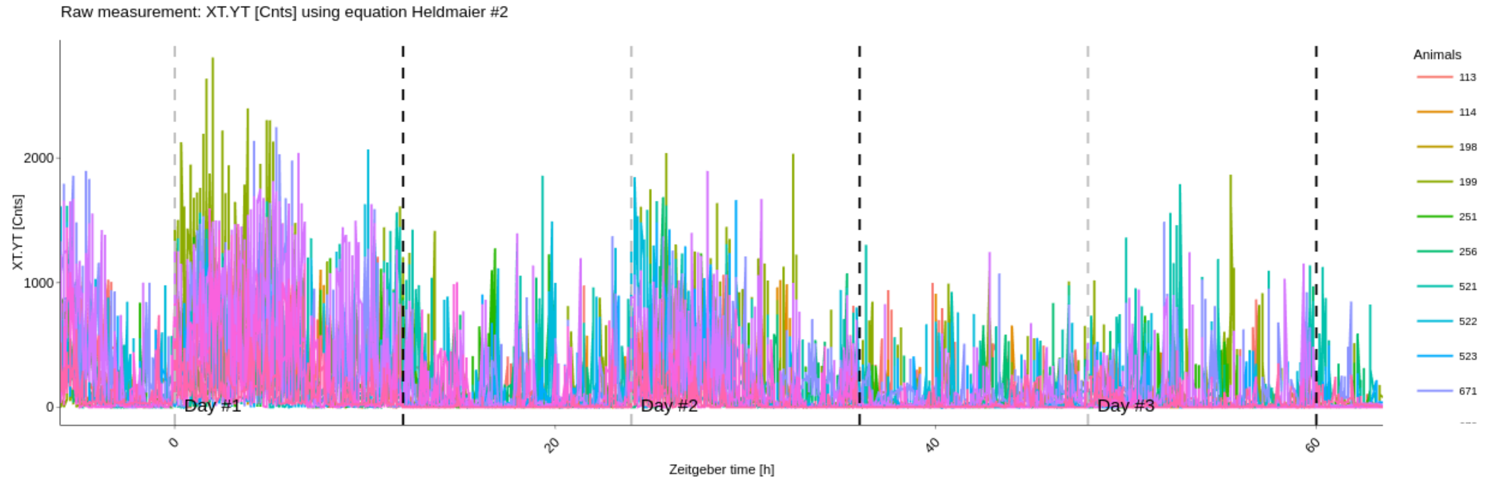

Odometering. Cumulative distance traveled in a metabolic cage per animal:

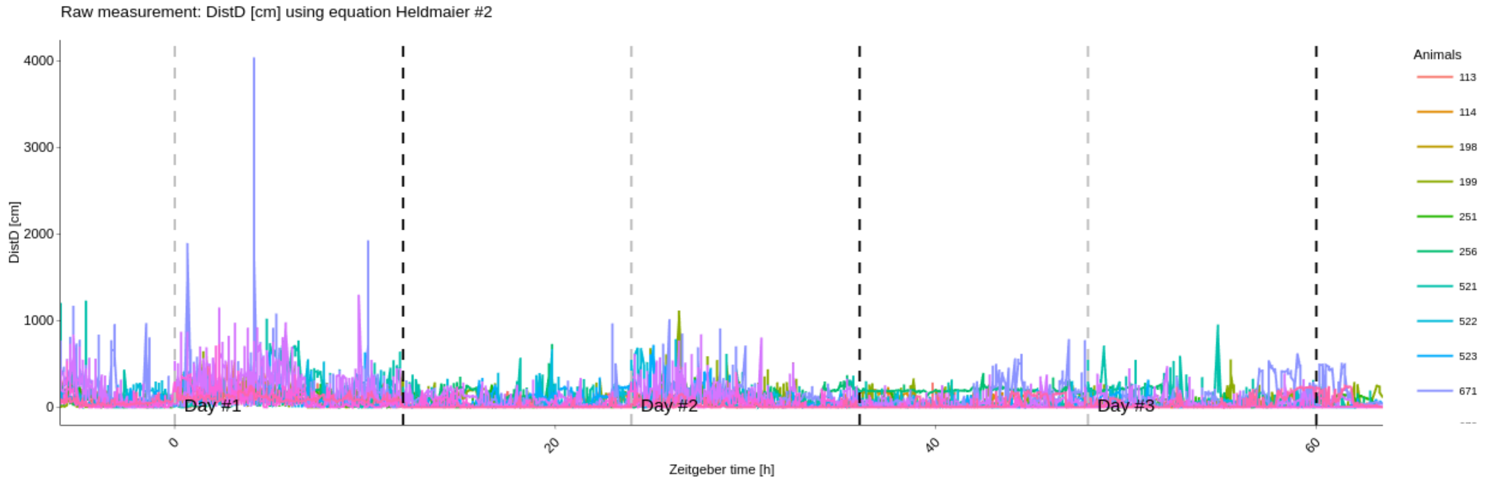

Feeding periods. Cumulative amount of food intake per animal:

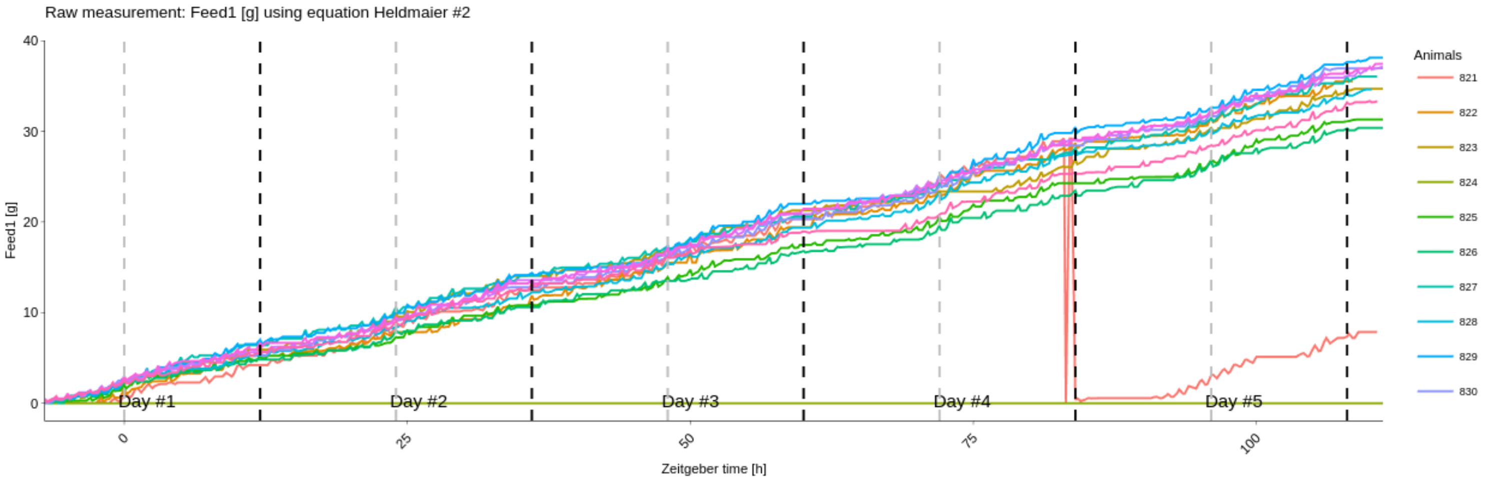

Drinking periods. Cumulative amount of water intake per animal:

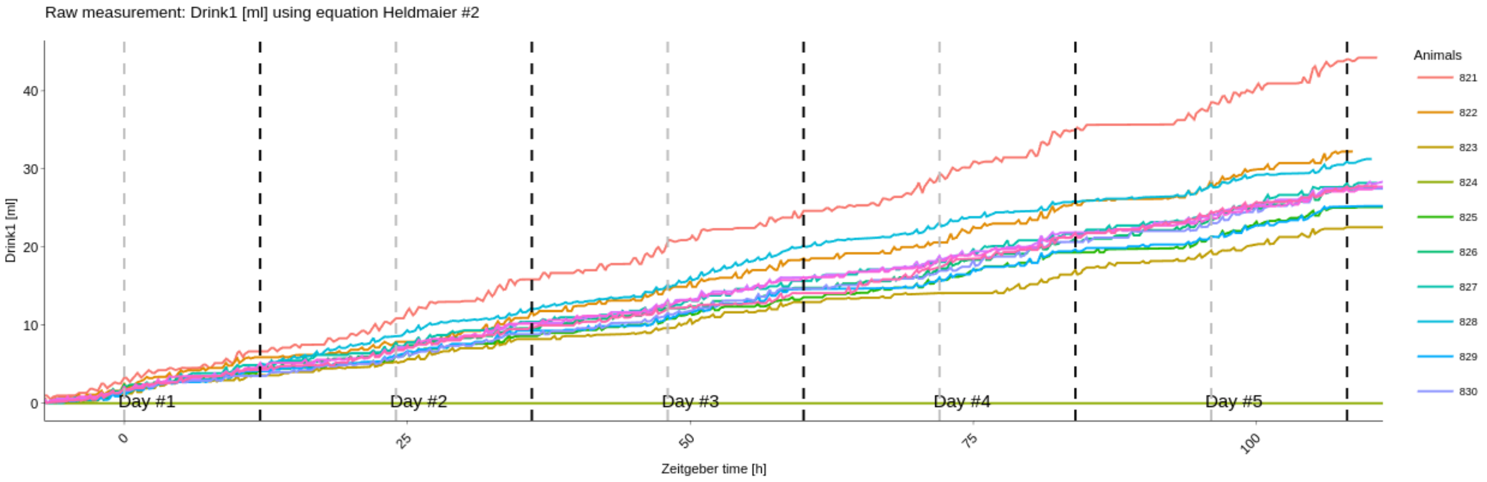

Body weight tracking. Current body weight (as determined by animals sporadically using the scale installed in the metabolic cage) over time per animal:

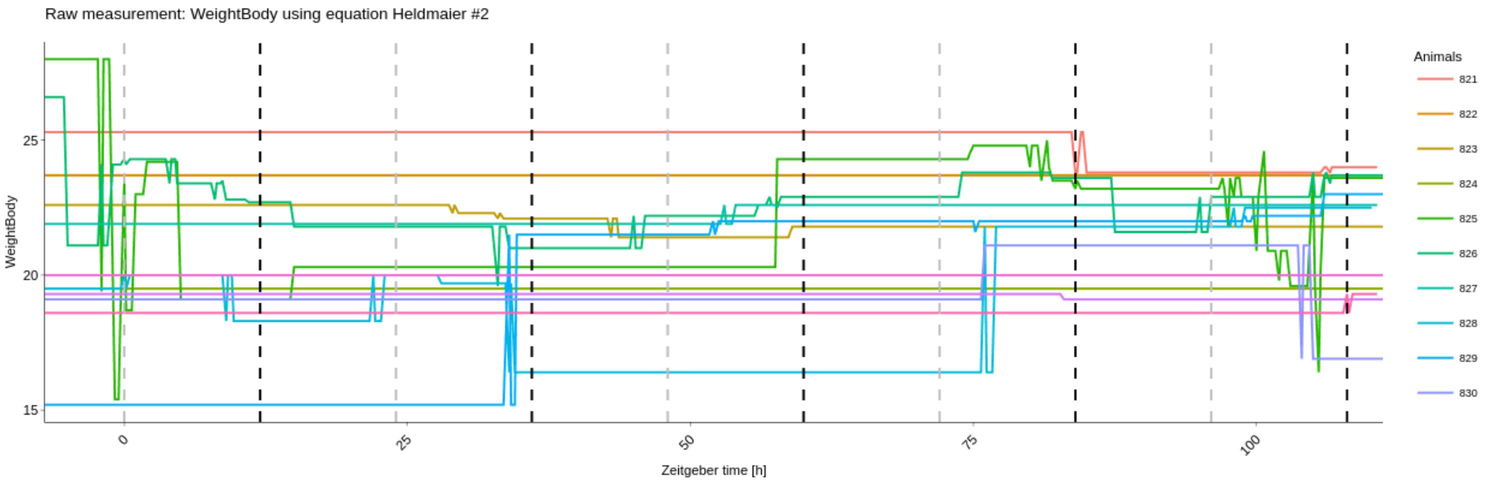

6.3 III. Dataset generation from the IMPC database

The International Mouse Phenotyping Consortium (IMPC) contains raw data from a variety of indirect calorimetry experiments for different genotypes (and knockouts) as well as metabolic phenotypes.

When experimenters lack specific indirect calorimetry experiments, and still wish to analyze a knockout experiment for a given genotype (e.g. Ucp1 or Adipoq), the IMPC database might become useful.

While there exists a programmatic access through the Solr/Apache REST API to the IMPC database, complete indirect calorimetry datasets cannot be retrieved directly. However indirect calorimetry datasets can be manually created from separate datasets stored in the IMPC databases, tracking O_2, CO_2, etc. and additional metadata.

To allow Shiny-Calorie users to easily compile indirect calorimetry datasets for their choice of genotype from the IMPC database, a Python package called `calorimetry-tools`, cf. Listing 1. The package automatizes the generation of indirect calorimetry datasets from the IMPC database and stores the result in the TSE 6.3 row-based format which is directly loadable in Shiny-Calorie. The Python package can also be called via `reticulate` from GNU/R directly from Shiny-Calorie, however a pre-assembly of indirect calorimetry datasets is recommended, since large data retrieval from the IMPC database will take some processing time.

```python
from calorimetry_tools import *

def convert_from_impc_to_tse_file(gene_symbol):
    """Wrapper to convert from IMPC dataset to a TSE file"""
    # retrieve calorimetry measurements for a given gene symbol
    measurements = get_measurements_for_gene_symbol(gene_symbol)

    # combine these calorimetry measurements and store as data frame
    df = combine_measurements_for_gene_symbol(gene_symbol, measurements)

    # convert and write df to TSE PhenoMaster v6 row format
    write_tse(df, gene_symbol, f"results/TSE_file_for_{gene_symbol}.csv")

if __name__ == "__main__":
    # Example gene symbols
    gene_symbols = ["Ucp1", "Adipoq"]

    for gene in gene_symbols:
        convert_from_impc_to_tse_file(gene)
```

Listing 1: Example code to generate indirect calorimetry datasets for gene symbols Ucp1 and Adipoq from the IMPC database. The `calorimetry-tools` package makes internally use of the Apache/Solr REST API provided by the IMPC. Package is available on Github: `https://github.com/stephanmg/calorimetry-tools`.

6.4 IV: Wavelet analysis to study ultradian rhythms

When high-frequency measurements (< 60 secs measurement interval) are available, the study of e.g. ultradian rhythms (URs) becomes possible. The study of URs has many important implications for unraveling the underlying cellular mechanisms of the complex behavioral patterns of e.g. instance Djun-

garian hamsters. We note, that in principle wavelet analysis can be applied also in cases when no high-frequency measurement data is available.

6.4.1 Mathematical foundations

To study ultradian rhythms we make use of the continuous wavelet transform (CWT). Contrary to the traditional Fourier transformation (FT), which assumes a stationary signal, CWT does allow for non-stationary signals. FT is used to reveal globally (over time) the magnitude of contributions of different frequency components. CWT, not assuming a stationary signal, can resolve changes in the contributions of frequency components time-dependent, i.e. ideal to reveal which frequencies are dominant at a given point in time.

More formally, let $x(t)$ be a time series signal representing, for example, metabolic rate of a mouse.

The CWT of $x(t)$ at scale s and translation τ is defined as:

$$W_x(s,\tau) = \frac{1}{\sqrt{s}} \int_{-\infty}^{\infty} x(t)\, \psi^* \left(\frac{t-\tau}{s} \right) dt$$

where:

- $\psi(t)$ is the mother wavelet,
- $\psi^*(t)$ is the complex conjugate of the mother wavelet,
- s is the scale parameter (the inverse to frequency),
- τ is the translation parameter or time shift.

For signal analysis in biological contexts, the mother wavelet is commonly chosen to be the Morlet wavelet:

$$\psi(t) = \pi^{-1/4} e^{i\omega_0 t} e^{-t^2/2}$$

where ω_0 is the central frequency.

The scale s can be converted to an ordinary Fourier period T by the relationship

$$T = \frac{s}{f_c}$$

where f_c is the center frequency of the chosen wavelet. When chosing the Morlet wavelet, the center frequency is commonly approximated by $f_c = \frac{\omega_0}{2\pi}$.

Wavelet power spectrum To measure the signal power at a given scale and time, one can calculate the wavelet power spectrum:

$$P_x(s,\tau) = |W_x(s,\tau)|^2$$

The wavelet power spectrum is a two-dimensional function of the scale (or period) and time which visualizes the rhythmic activity at different frequencies over time.

Global wavelet spectrum While the wavelet power spectrum provides detailed information of the frequency distribution at a given time, the global wavelet spectrum provides the overall contribution of each scale (or period) across the total time T of the signal and is defined by:

$$G(s) = \frac{1}{T} \int_0^T |W_x(s,\tau)|^2 d\tau$$

6.4.2 Example application

Shiny-Calorie allows to study high-frequency recordings from the TSE Calobox indirect calorimetry system (single chamber) and to conduct wavelet analysis (as described previously in [10]) to detect URs, cf. Figs. S4-S5. Depicted is the power spectrum and the major significant frequency components based on the oxygen consumption which is proportional to the metabolic rate. Shiny-Calorie allows all calculated, derived and raw quantities to be analyzed by wavelet analysis. Interpolation to regularly-spaced time grids, pre-smoothing with LOESS (locally estimated scatterplot smoothing), Savitzky-Golay filtering, or correction for the delay of the measured gas exchange signals (properties of the measurement chamber) through z-transformation [11] (instantaneous metabolic rate) are subject to configuration by users when using TSE Calobox files, Shiny-Calorie provides reasonable defaults from literature. Significant periods are marked in the periodogram with red circular markers.

6.5 Estimation of resting metabolic rate

To estimate the resting metabolic rate, we apply the following procedure.

We define the following variables:

Oxygen and carbon dioxide concentrations are typically recorded at discrete time points, e.g. 3, 5 or 10 minutes apart and not monitored continuously, thus we denote the time points of measurement by $t_k = k \cdot \Delta t$, where Δt is the interval length and k is the number of intervals which have passed. We define the following variables:

- $x_{\mathrm{O}_2}(t_k)$: Oxygen concentration at time t_k
- $x_{\mathrm{CO}_2}(t_k)$: Carbon dioxide concentration at time t_k and
- $t_k \in \{1, 2, \ldots, \lceil \frac{T}{\Delta t} \rceil\}$: Time points of measurement

We fix $\Delta t > 0$ and define a sliding window of length w (i.e., a multiple s of the spacing of the time-grid) as $s \times \Delta t$ minutes), yielding a total of:

$$K = T - w + 1$$

sliding windows.

For each window $k \in \{1, \ldots, K\}$, the set of time indices is:

$$\mathcal{W}_k = \{t_k, t_{k+1}, \ldots, t_{k+s-1}\}$$

Interval Subdivision

Each sliding window $\mathcal{W}_k$ contains $m = \left\lceil \frac{w}{\Delta_t} \right\rceil$ measurements.

For a chosen sub-interval length $l \in \{1, 2, ..., m-1\}$ we create a subdivisions of the current sliding window $\mathcal{W}_k$ as the consecutive sub-intervals:

$$\mathcal{I}_{k,l} = \{(t_{k+i}, t_{k+i+l}) | i = 0, 1, ..., m-l-1\}$$

Coefficient of Variation

For each of the sub-intervals $\mathcal{I}_{k,l}$, compute the mean and standard deviation:

$$\mu_{O_2}^{k,i} = l^{-1} \sum_{\mathcal{I}_{k,i}} x_{O_2}(t), \quad \sigma_{O_2}^{k,i} = \sqrt{l^{-1} \sum_{\mathcal{I}_{k,l}} \left(x_{O_2}(t) - \mu_{O_2}^{k,i} \right)^2}$$

$$\mu_{CO_2}^{k,i} = l^{-1} \sum_{\mathcal{I}_{k,i}} x_{CO_2}(t), \quad \sigma_{CO_2}^{k,i} = \sqrt{l^{-1} \sum_{\mathcal{I}_{k,l}} \left(x_{CO_2}(t) - \mu_{CO_2}^{k,i} \right)^2}$$

The coefficient of variation (CV) is:

$$\mathrm{CV}_{O_2}^{k,i} = \frac{\sigma_{O_2}^{k,i}}{\mu_{O_2}^{k,i}}, \quad \mathrm{CV}_{CO_2}^{k,i} = \frac{\sigma_{CO_2}^{k,i}}{\mu_{CO_2}^{k,i}}$$

Find the intervals (k_1, k_2) which have the minimum CV for the O_2 and CO_2 component:

$$\mathrm{CV}_{O_2}^{k_1} = \min_{i=1,\ldots,N} \mathrm{CV}_{O_2}^{k,i}, \quad \mathrm{CV}_{CO_2}^{k_2} = \min_{i=1,\ldots,N} \mathrm{CV}_{CO_2}^{k,i}$$

We chose the measurement t'_k according to the the minimum CV:

$$k' = \arg\min_{k_1,k_2} (\mathrm{CV}_{O_2}^{k_1}, \mathrm{CV}_{CO_2}^{k_2})$$

Assuming flow rates of V_{O_2}, V_{CO_2} are available at t'_k, the heat production (HP) at t'_k is defined as:

$$\mathrm{HP}(t_k) = (3.941 \cdot V_{O_2}(t'_k) + 1.106 \cdot V_{CO_2}(t'_k))$$

Summing over all windows yields the resting metabolic rate (RMR):

$$\mathrm{RMR} = \sum_{k=1}^{K} \mathrm{HP}(t_k)$$

The non-RMR contribution is defined as the difference between the total energy expenditure and the resting metabolic rate.

6.6 Comparison of energy expenditure equations

We found that the caloric equivalents obtained with the six different equations only deviate marginally from each other. This is illustrated by plotting the positive linear correlation of the caloric equivalent with the respiratory exchange ratio, as reproduced from a previous review (Evan and Nadkarni, 2012). At RER of 0.7, the caloric equivalent of $\approx$ 19.5 kJ x L O_2 is lowest, but rises steadily with increasing carbohydrate oxidation and attains $\approx$ 21 kJ x L O_2 at RER of 1.0 (Supplemental Figure S6). Comparing deviations of the caloric equivalents calculated as a percentage of the mean, the most extreme deviation is $< 2\%$, with three equations showing increasing (Heldmaier, Elia, Lusk) and three showing decreasing (Ferranini, Weir, Brower) deviations with increasing RER (Supplemental Figure S7).

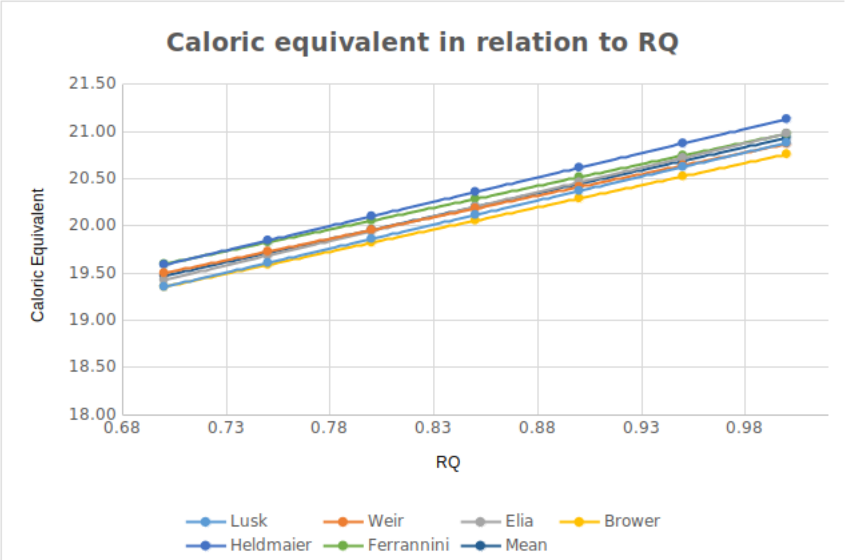

Supplementary Figure S6: Comparison of RQ and caloric equivalent for the different energy expenditure equations

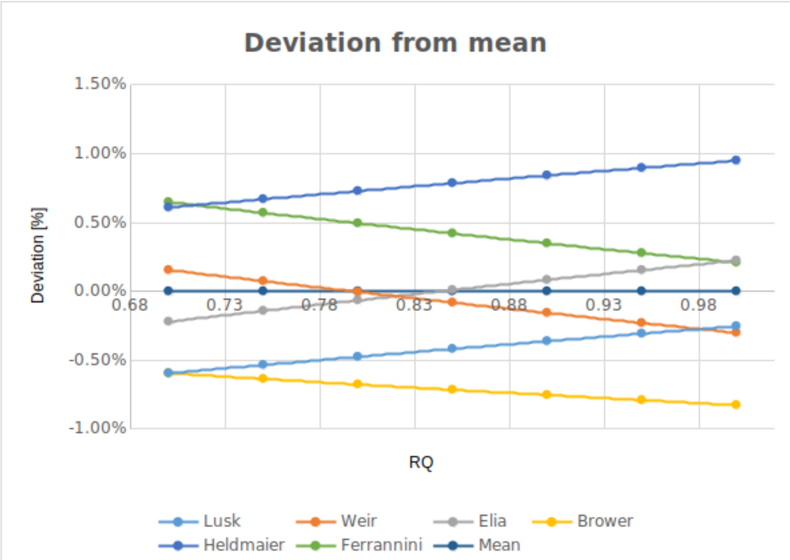

Supplementary Figure S7: Deviations from means for caloric equivalents and RQs calculated for the different energy expenditure equations

These slight differences in RER sensitivity stimulated us to compare EE with all six equations for the ADKO data set, where not only typical day-night RER fluctuations are apparent, but also a clear genotype difference in metabolic flexibility can be observed. ADKO mice show a dampening in the day-night amplitude of RER (Figure 3). In such data sets, the RER sensitivity may affect the EE calculations and thus bias the detection of genotype effects. We addressed the RER sensitivity of EE calculations in three ways.

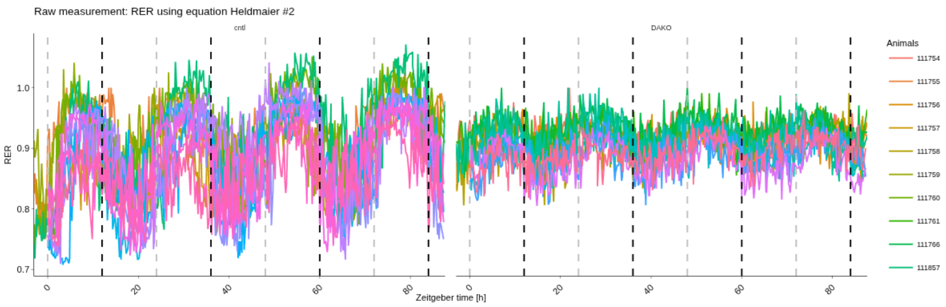

Supplementary Figure S8: Differences in RER patterns over time for a double knockout study (ADKO) and the wild type (CTRL)

First, the plot of EE in kJ/h by RER showed a sigmoidal positive association of EE with RER for ADKO and CTRL (Supplemental figure S9). This is in line with the expectation that carbohydrate fuel utilization increases with higher energy turnover. This relationship was largely the same for five out of six equations, with the Lusk equation delivering significantly higher EE values at low RER for CTRL and at intermediate RER for ADKO. The Brouwer Second, EE (kJ/h) calculated with all six equations was plotted over three days of continuous measurements for ADKO and CTRL (Supplemental Figure S10). Statistical evaluation revealed a significant genotype effect during the photophase for EE values calculated by four equations (Heldmaier, Weir, Ferranini, Brouwer), but not for Lusk and Elia. This single observation on the first day of indirect calorimetry is most likely not biologically relevant, but suggests that the different RER sensitivity of the equations may matter.

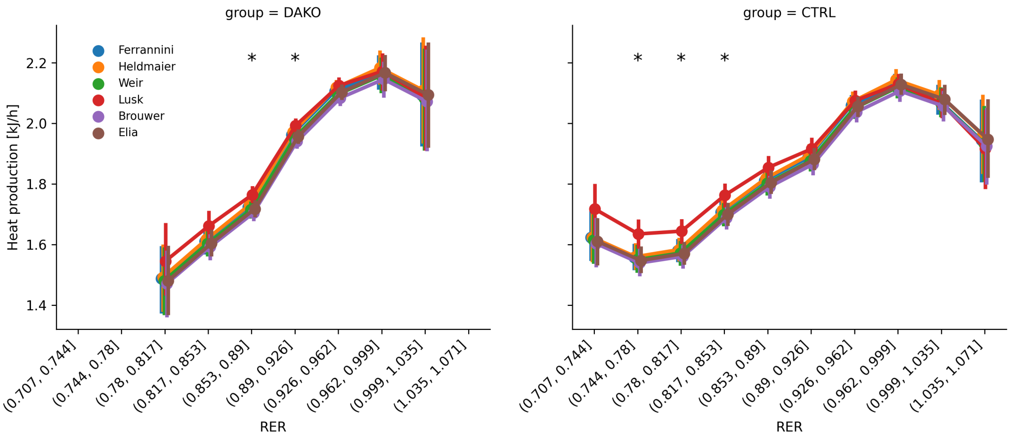

Supplementary Figure S9: RER sensitivity for heat production (in intervals) binned by RER for different EE equations

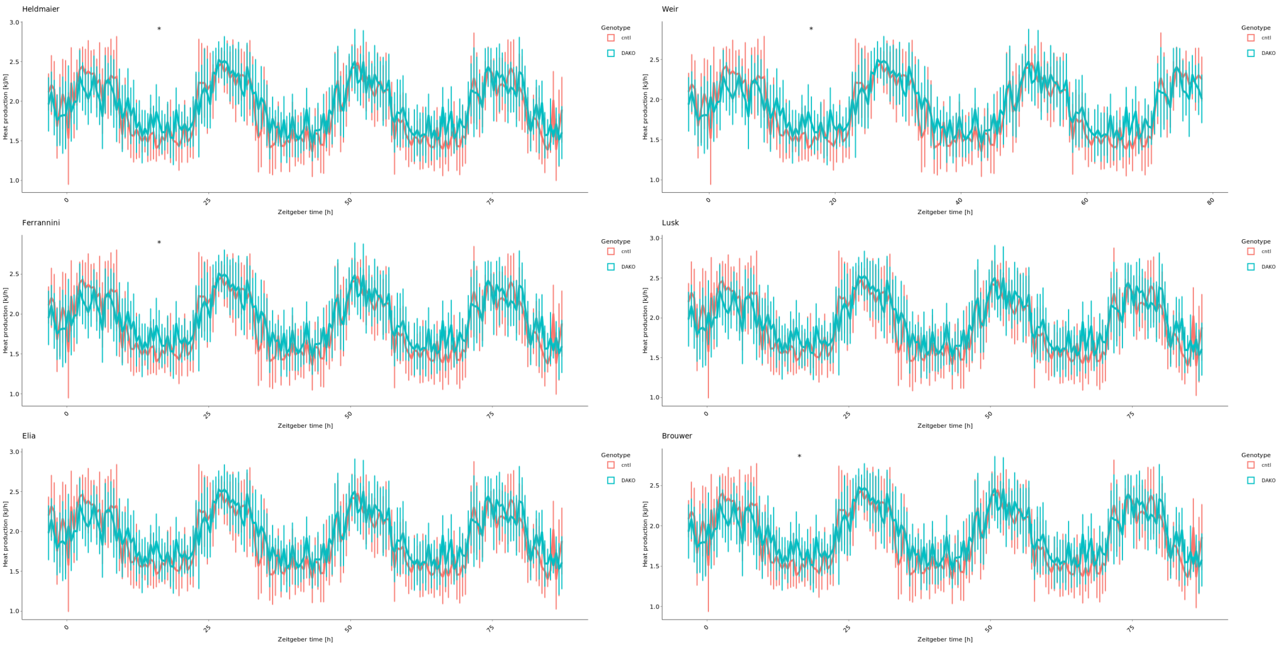

Supplementary Figure S10: RER sensitivity for heat production over time for different EE equations

Third, we created scatter plots of pairwise comparisons for all six equations (Supplemental Figure S11). The EE values calculated by the Lusk equation were lower when compared to the values calculated by all other equations. This discrepancy tended to increase at high EE values. All other pairwise comparisons revealed more or less identical EE values.

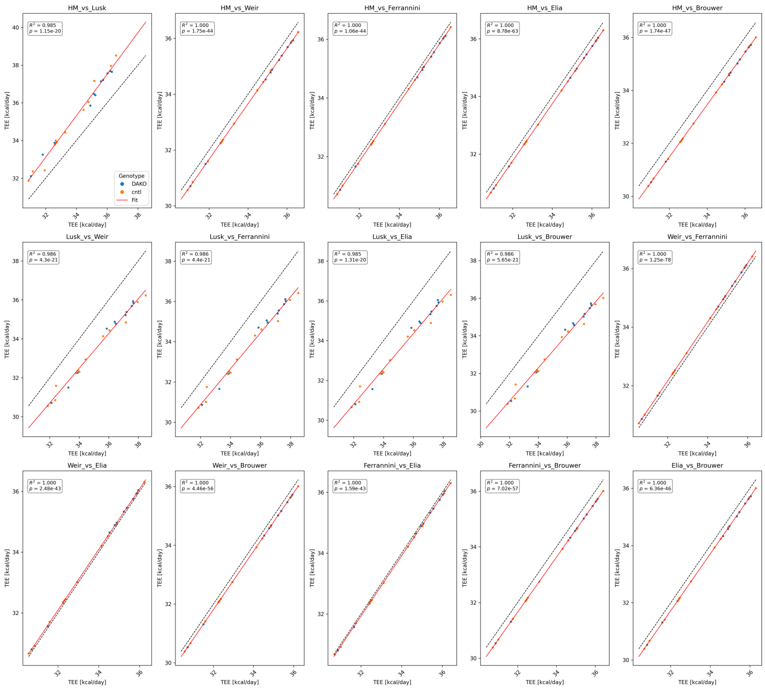

Supplementary Figure S11: Scatter plots for Weir, Heldmaier and Lusk. Genotypes (ADKO and control) are colored accordingly.

Based on this analysis, we conclude that the equations by Brouwer, Ferranini, Heldmaier, and Weir are equally suitable to calculate EE, whereas we have some reservations about applying the equations by Elia and Lusk.

References

[1] L. Seep et al. From Planning Stage To FAIR Data: A Practical Metadatasheet For Biomedical Scientists. *Sci Data*, 11, 2024.

[2] P. C. Even and N. A. Nadkarni. Indrect calorimetry in laboratory mice and rats: principles, practical considerations, interpretation and perspectives. *Am J Physiol Regul Integr Comp*, 303(5), 2012.

[3] G. Heldmaier. Metabolic and thermoregulatory responses to heat and cold in the djungarian hamster. *J. Comp. Phys.*, 1975.

[4] J. B. de V. Weir. New methods for calculating metabolic rate with special reference to protein metabolism. *The Journal of Physiology*, 109(1-2):1–9, 1949. doi: https://doi.org/10.1113/jphysiol.1949.sp004363. URL https://physoc.onlinelibrary.wiley.com/doi/abs/10.1113/jphysiol.1949.sp004363.

[5] E. Ferrannini. The theoretical bases of indirect calorimetry: A review. *Metabolism*, 37(3):287–301, 1988. ISSN 0026-0495. doi: https://doi.org/10.1016/0026-0495(88)90110-2. URL https://www.sciencedirect.com/science/article/pii/0026049588901102.

[6] G. Lusk. *The Elements of the Science of Nutrition.* Sanders, Philadelphia, PA, 1928.

[7] M. Elia and G. Livesey. Energy Expenditure and Fuel Selection in Biological Systems: The Theory and Practice of Calculations Based on Indirect Calorimetry and Tracer Methods. In *Metabolic Control of Eating, Energy Expenditure and the Bioenergetics of Obesity.* S.Karger AG, 09 1992. ISBN 978-3-8055-5595-1. doi: 10.1159/000421672. URL https://doi.org/10.1159/000421672.

[8] E. Brouwer. Report of sub-committee on constant and factors. *Energy metabolism*, 11:441–443, 1965.

[9] M. Klingenspor, T. Fromme, and S. Maurer. Ablation of uncoupling protein 1 causes opposing changes of glucose uptake in brown and brite fat compatible with regulation of Glut 4 gene expression. *The FASEB Journal*, 34(S1), 2020.

[10] G. Heldmaier. Multiple ultradian rhythms of metabolism, body temperature and activity in djungarian hamsters. *J Comp Physiol B 194*, page 501–518, 2024. doi: https://doi.org/10.1007/s00360-024-01569-x.

[11] G. Bartholomew, D. Vleck, and C.M. Vleck. Instantaneous measurements of oxygen consumption during pre-flight warm-up and post-flight cooling in sphingid and saturniid moths. *J. Exp. Biol.*, pages 17–32, 1981.
